# How do sports participation and sports settings influence the mental health of children and adolescents? A systematic review of qualitative studies

**DOI:** 10.1186/s12889-025-25916-x

**Published:** 2025-12-18

**Authors:** Linda Ooms, Wikke van Stam, Vassilis Sevdalis, Marie Overbye, Beatrix Algurén, Sandra Heck, Aurélie Van Hoye

**Affiliations:** 1 Department of Sport, Physical Activity & Health, Mulier Institute, Herculesplein 269, Utrecht, 3584 AA The Netherlands; 2 Department of Food and Nutrition and Sport Science, University of Gothenburg, Läroverksgatan 5, Gothenburg, 41120 Sweden; 3 Department of Nutrition, Exercise and Sports, University of Copenhagen, Nørre Allé 51, Copenhagen N, 2200 Denmark; 4 LUNEX - International University of Health, Exercise & Sports, 50, Avenue du Parc Des Sports, Differdange, L-4671 Luxembourg; 5 Faculté des Sciences du Sport, UMR1319 INSPIIRE, Université de Lorraine, 30 rue du Jardin Botanique, CS 30156, Villers-lès-Nancy, 54600 France; 6 Physical Activity for Health research center, Physical Education and Sport Sciences Department, University of Limerick, Limerick, V94TP9X Ireland

**Keywords:** Mental health, Sports participation, Sports setting, Children, Adolescents, Socio-ecological model, Mechanisms, Factors

## Abstract

**Background:**

Promoting mental health through sports participation may be a promising strategy to support the mental health of children and adolescents. This systematic review investigated the perceptions of children and adolescents and of stakeholders close to this target group (e.g., teammates, coaches, parents) on 1) how sports participation influences the mental health of children and adolescents; and 2) factors in sports settings influencing this relationship.

**Methods:**

A systematic review of qualitative studies was performed. Six electronic databases (CINAHL, PsycInfo, PubMed, Scopus, SPORTDiscus, Web of Science) were searched for relevant studies from inception to 21 August 2024. Out of 1,001 identified records, 25 studies were included. Study quality was assessed using the CASP-tool. Thematic analysis was performed to synthesize findings, which were structured according to the socio-ecological model. Results are presented according to three main topics: 1) mental health outcomes (intrapersonal level of socio-ecological model); 2) individual mechanisms (intrapersonal level); and 3) factors in the sports setting (interpersonal, and organizational and community levels).

**Results:**

Sports participation influenced various mental health outcomes, including emotions, self-confidence and self-esteem, levels of stress, and resilience, both positively and negatively. Five key individual mechanisms were identified through which sports participation influenced mental health: distraction and focus, performance, personal development, purpose and structure in life, and a sense of belonging. Additionally, interpersonal factors, such as the influence of coaches, teammates and peers, family members, and (team) professionals, appeared important in the relationship between sports participation and mental health. The broader social climate (organizational and community levels) also influenced this relationship. Based on the socio-ecological model and the review findings, a theoretical model was developed illustrating these interconnections.

**Conclusions:**

The findings confirm the potential mental health benefits of sports participation among children and adolescents, which are largely influenced by interpersonal factors. However, some negative mental health outcomes were also identified and may be underreported. These insights can guide targeted interventions to enhance youth mental health through sports participation, aligned with the proposed theoretical model. Future research should refine this model, assess its applicability across populations, developmental stages, sports activities, and settings, and explore its links with sport drop-out.

**Supplementary Information:**

The online version contains supplementary material available at 10.1186/s12889-025-25916-x.

## Background

Mental health is increasingly recognized as an integral component of general health and well-being. The World Health Organization (WHO) defines mental health as ‘a state of mental well-being that enables people to cope with the stresses of life, realize their abilities, learn and work well, and contribute to their communities.’ [1] This definition emphasizes that mental health extends beyond the mere absence of mental disorders, encompassing positive attributes such as resilience, effective functioning, and social contribution. Mental health exists along a continuum, ranging from optimal states of mental well-being to debilitating states of suffering and emotional pain. Although mental health is not defined by the presence or absence of mental disorders, individuals experiencing such disorders are more likely to report lower levels of mental well-being [1].

In recent years, increased attention has been given to mental health by policy makers, researchers, and practitioners alike. Several findings worldwide indicate that mental health problems are becoming highly prevalent [1]. Children and adolescents represent a particularly important target group, as mental health is fundamental to every aspect of their development. High levels of mental health support their ability to learn effectively, form and maintain healthy relationships, regulate emotions, and adapt to life’s challenges. A strong foundation of mental health enables children and adolescents to thrive across key environments, such as at home, in school, and within their communities. Moreover, safeguarding mental health during these formative years establishes the groundwork for lifelong mental health, and contributes to healthier, more resilient, and productive adulthoods, ultimately strengthening the overall well-being of communities and societies [1, 2]. Simultaneously, childhood and adolescence are critical stages in human development, characterized by profound biological (including hormonal and neural), psychological, and social changes. Individuals go through different developmental milestones, which can make them more vulnerable to mental health problems [1–6]. Rapid social, technological, and economic changes in society, such as pervasive smartphone and social media use, academic pressures, and increasing inequalities, pose additional challenges to young individuals’ mental health. These risks are further compounded by global stressors, including climate change and pandemics [1–4]. In this regard, research shows that the Covid-19 pandemic, and related social isolation and fears, exacerbated mental health problems among children and adolescents [1, 3, 7–10]. Globally, the prevalence of clinically elevated depression (25.2%) and anxiety symptoms (20.5%) has, for example, doubled among this population group in comparison with pre-pandemic estimates (12.9% for depression and 11.6% for anxiety) [9]. Children and adolescents with poor mental health may be more vulnerable to stigma, social exclusion, educational difficulties, risk-taking behaviors, and physical ill-health [1, 2, 11–16]. Moreover, poor mental health in childhood or adolescence is associated with impaired mental health and an increased likelihood of mental disorders in adulthood [1, 2, 17–19]. Therefore, there is a need for affordable, effective, and feasible strategies to promote, protect, and restore the mental health of this population group [1, 3, 7, 15, 16]: promoting mental health through sports participation can be one such strategy, of distinct and potential interest.

Sport is a specific form of physical activity which involves physical effort and/or physical skill and can be undertaken individually or with others. Participants adhere to a common set of rules or expectations; and there is also the possibility to compete against others [20, 21]. A growing body of research indicates that sports participation is associated with a range of mental health benefits for children and adolescents. It may help prevent the onset of mental health difficulties, as it has been linked to lower levels of anxiety and depressive symptoms [21–25]. Moreover, sports participation can promote mental health in other ways, for example, by enhancing self-confidence and self-esteem, fostering emotional regulation, stimulating social interaction, and supporting the development of social skills [21–23, 25]. In this sense, there is some evidence that team sports may provide enhanced mental health outcomes, compared with individual sports activities, due to the social nature of the participation [21–23, 25]. Accordingly, the interest in the promotion of mental health through sports participation and in sports settings has increased in recent years within research, policy, and (sports) practice [26–28]. In Europe, 66% of adolescents (15–24 years) participate in sports on a weekly basis [29]. For children, sports participation rates are suggested to be even higher [30]. Therefore, the mental health benefits of sports participation can be potentially widespread. However, young individuals who feel mentally unhealthy are less likely to participate in sports than those that feel mentally healthy [31, 32]. Moreover, sports participation can occasionally have negative mental health impacts as well, such as performance anxiety, increased stress, and burn-out [23, 25, 33]. Therefore, mental health benefits may depend on the experience of sports participation and the context in which it takes place [6, 20–22, 28].

The influence of sports participation on mental health has been conceptualized through the socio-ecological model [6, 20, 21], which situates individual sport experiences within multiple, interacting levels of influence. Beyond the intrapersonal level (i.e., factors at the level of the child or adolescent), these include the interpersonal level (e.g., influence of teammates, coaches, parents), the organizational level (e.g., sports club’s policies), the community level (e.g., social norms, characteristics of the physical environment), and the policy level (e.g., local and national policies on sports participation and mental health) [6, 20, 21, 34, 35]. Factors at each level can facilitate or hinder positive mental health outcomes, by both shaping the sport experience and its impact on mental health. Building on this model, Eime et al. (2013) proposed the ‘Health through Sport’ model, which emphasizes that individual physical, mental, and social health outcomes emerge from interactions between the sports participant and the broader sports environment [21]. Eather et al. (2023) extended this with the ‘Mental Health through Sport’ model, by identifying several mechanisms that may explain how sports participation enhances mental health [20]. These include neurobiological (e.g., brain structure and function changes), psychosocial (e.g., opportunities for mastery), behavioral (e.g., improved sleep), and social mechanisms (e.g., development of social relationships and social support) [20, 36, 37]. Together, these socio-ecological models offer a foundation for understanding how, for whom, and under what conditions sports participation may promote mental health. They highlight that benefits arise from complex, multi-level interactions among intrapersonal, interpersonal, organizational, community, and policy factors. Nevertheless, further research is required to clarify the specific mechanisms and contextual factors that influence mental health outcomes through sports participation. This need applies to sports participation in general, across different stages of development, and particularly among children and adolescents [20, 21, 24, 36, 37].

The aforementioned research on sports participation and mental health of children and adolescents is mostly based on quantitative approaches [21–25]. While quantitative research is valuable for identifying patterns, associations, and generalizable trends, it often lacks the depth required to capture the nuanced experiences and contextual factors that influence young individuals’ engagement in sport and its effects on mental health. In the last decade, however, a growing number of qualitative studies have been conducted in this area, e.g., [33, 38, 39]. Qualitative research adds significant value by providing insight into the meanings, perceptions, and lived experiences of children and adolescents, thereby enabling a deeper understanding of how and why sports participation influences their mental health. For example, it can explore how sports participation affects self-confidence and identify the specific factors within sports settings that shape this relationship. By reviewing and thematically synthesizing qualitative studies, this study integrates diverse perspectives to generate a more comprehensive understanding of the mechanisms and factors linking sports participation and mental health. Such a synthesis allows a richer interpretation of the complex, multi-level processes involved, producing insights that extend beyond the findings of individual studies and complement evidence derived from quantitative research [40, 41].

Therefore, we conducted a systematic review of qualitative studies focusing on the following research questions: 1) How does sports participation influence the mental health of children and adolescents?; and 2) What factors in sports settings influence this relationship? In this review, we focused on perceptions of children and adolescents themselves and of stakeholders close to this target group (e.g., teammates, coaches, parents). In addition, we focused both on positive and negative influences on mental health, as well as facilitating and impeding factors. By doing so, we aimed to provide a more in-depth understanding of the perspectives of individuals or groups within a sport-related context, so that we can increase the potential of health promotion and health intervention efforts aiming at improving the mental health of children and adolescents through sports participation.

## Methods

This review was conducted and reported in accordance with the PRISMA (Preferred Reporting Items for Systematic reviews and Meta-Analyses) guidelines and checklist [42, 43] (see Additional File 1). Additionally, the ENTREQ (Enhancing Transparency in Reporting the synthesis of Qualitative research) statement for reporting qualitative syntheses was applied [44]. The review protocol was registered in PROSPERO on 5 August 2024 (registration number: CRD42024576737).

### Eligibility criteria

The eligibility criteria used to select relevant studies are outlined below using the PICO-framework. This method structures research questions by focusing on four key components: the Population of interest (P), the Intervention or exposure being studied (I), the presence or absence of a Comparator group (C), and the desired Outcomes (O). In addition, eligibility criteria related to the types of studies were included.

#### Population of interest

Studies were included if the population of interest was children (5–12 years) and/or adolescents (13–25 years). For adolescents, a broad age range was chosen, because this aligns with recent research showing that important developmental changes (e.g., biological, psychological, social) that start in puberty end in the mid-20s [3–5]. Perspectives on the research questions could come from the population of interest, but also from people close to the target group, such as teammates, coaches, and parents. Studies were excluded if they focused on individuals outside the age range 5–25 years or on individuals with an intellectual disability.

#### Intervention/exposure

Studies were included if they addressed participation in sports and were conducted in a sports setting. Based on existing definitions in the literature [20, 21], ‘sport’ was defined as: ‘an activity requiring physical effort and/or physical skill that can be undertaken individually or with others, where participants adhere to a common set of rules or expectations; there is also the possibility to compete against others.’ ‘Sports settings’ were defined as: ‘settings where sports activities are performed, whether it is a training session, competition, or game, ranging from (un)organized community sports (i.e., settings focused on fun and enjoyment whilst playing sport) [28] to professional sports (i.e., settings focused on competition where elite athletes often receive payment).’ Studies were excluded if they focused on: a) ‘physical education’—defined as ‘a sequential, developmentally appropriate educational experience that engages students in learning and understanding movement activities that are personally and socially meaningful, with the goal of promoting healthy living’ [45]; b) ‘exergaming’—defined as ‘technology-driven physical activities, such as video game play, that require participants to be physically active or exercise in order to play the game’ [46]; c) sports activities performed in a clinical health care setting.

#### Comparator

Not applicable.

#### Outcome

Studies were eligible if they provided data on (changes in) mental health outcomes in relation to sports participation and/or on factors in the broader sports setting influencing this relationship. ‘Mental health’ was defined as (see also: background): ‘a state of mental well-being that enables people to cope with the stresses of life, realize their abilities, learn and work well, and contribute to their communities. Mental health is an integral component of health and well-being and is more than the absence of mental disorders’ [1].

#### Types of studies

Eligible studies were original qualitative studies (e.g., research based on interviews, focus groups, case studies), published in a peer-reviewed journal, and in English language. Mixed-methods studies were also included when the qualitative research results were presented separately. The following publication types were excluded: grey literature, books, book chapters, editorials, conference proceedings, master theses, dissertations, literature reviews, and governmental/nongovernmental organization reports.

### Information sources

Six electronic bibliographical databases including CINAHL (through EBSCOhost), PsycInfo (through Ovid), PubMed, Scopus, SPORTDiscus (through EBSCOhost), and Web of Science (through Clarivate) were searched for relevant studies from inception of the database to 21 August 2024. In addition, the reference lists of included studies and relevant reviews identified through the searches were scanned. This last step did not result in additional studies.

### Search strategy

Literature searches were developed using text words and, where possible, Medical Subject Headings (MeSH) related to: 1) the population of interest (children and adolescents); 2) the intervention/exposure (sport); 3) the outcome (mental health); and 4) the types of studies (qualitative research). This resulted in four search blocks or strings of words. These strings were combined with the Boolean operator ‘AND’: String 1 AND String 2 AND String 3 AND String 4. The searches in the databases were restricted to title and abstract. No additional filters were applied in the databases. See Additional file 2 for the search strategy per database.

### Study selection

The search results in each database were exported to one common file in EndNote version 21.5. Duplicate records and records of ineligible publication type (e.g., books, conference proceedings) were removed. In the next step, the remaining records were exported to the software program Rayyan (https://www.rayyan.ai/) to facilitate the study selection process among various independent reviewers. Specifically, two authors (LO, WvS) independently screened the titles and abstracts against the inclusion and exclusion criteria. Disagreements were resolved by discussion. Subsequently, full-text articles were obtained for all studies that met the inclusion criteria or when there was any uncertainty. Each full-text article was independently examined by two authors to decide whether it met the inclusion criteria, whereby articles were divided between two pairs of authors (LO & VS and WvS & MO). Any discrepancies were resolved by discussion between authors (within pairs).

### Data extraction and analysis

Initially, each article was read. Data extraction started with extracting the general characteristics of the study: author(s), publication year, country, study aims, study design, method of data collection, method of data analysis, definition of mental health, theoretical framework used, participants’ characteristics, sport discipline, characteristics of sports participation, and characteristics of the sports setting. For the first five articles (20%), these data were extracted independently by two authors (LO, WvS) using a standardized data-extraction form (in Excel) developed and pre-tested for the present review. Data extraction was compared, discrepancies were discussed, and data extraction was refined. After that, one author (LO) extracted the data of the remaining studies. Nonetheless, all sections of each article were read by two authors (LO, WvS).

Secondly, the results section (and discussion section where results and discussion were combined) of each article were analyzed with the support of MAXQDA 24 (release 24.7.0) software. Thematic analysis was performed to analyze and synthesize the data regarding the research questions [47]. An inductive approach was taken and initially LO and WvS independently conducted line-by-line coding of text. Both author interpretations and participant quotes were coded. After initial coding, codes were compared between the two authors and refined when necessary. Subsequently, codes were organized into descriptive sub-themes and main themes in relation to the research questions. This process of thematic synthesis was also performed by taking into consideration the socio-ecological model of health promotion [6, 20, 21, 34, 35], which provided guidance for organizing the main themes. When performing this synthesis, the research questions were divided into three main topics. Research question 1 was divided in two sub topics: 1) *Changes in mental health outcomes*: i.e., experienced changes in mental health outcomes within children or adolescents, for example, increased self-confidence through sports participation (intrapersonal level of the socio-ecological model); and 2) *Individual mechanisms*: i.e., described mechanisms at the intrapersonal level of how sports participation influences mental health, such as through distraction from daily stressors (also intrapersonal level of the socio-ecological model). The results of research question 2 were organized under the main topic: *Factors in the sports setting*: i.e., factors in the broader sports setting that influence the relationship between sports participation and mental health, such as support from teammates. Regarding this topic, factors mainly applied to the interpersonal level of the socio-ecological model. Factors related to the social climate were organized on the organizational and community levels. These levels were combined because children and adolescents participated in sports both within sports organizations (e.g., a sports club) and the broader community (e.g., a local sports event). Factors at the policy level did not appear to be important themes across the included studies.

Within each main topic, main themes—and in some cases, sub-themes—were organized. While subgroup comparisons were not the main focus of the study, we explored whether certain main themes or sub-themes were characteristic of specific subgroups. Subsequently, the results were discussed with all authors. This only led to minor adjustments in the sub-themes and the sorting of sub-themes in main themes. After that, the coding of all articles was again checked by both reviewers (LO, WvS).

### Critical appraisal of studies

The Critical Appraisal Skills Programme (CASP) Qualitative Studies Checklist was used to appraise the quality of the studies [48]. The checklist consists of 10 items or questions, for example, about the clarity of the study aims, the appropriateness of the qualitative research design, and the rigor of the data analysis. Each item in the tool is rated as ‘yes’, ‘no’ or ‘can’t tell’. This checklist has been used previously in other systematic reviews on similar topics [20, 49]. The critical appraisal was conducted independently by two authors (LO, WvS). Any discrepancies in rating were resolved by discussion. No studies were excluded based on quality, as there is limited evidence to suggest this is beneficial [50, 51]. However, quality assessment allows for transparency and enhances our understanding of the contribution made by each study to the findings and conclusions of this review.

## Results

### Characteristics of the included studies

**Fig. 1:**
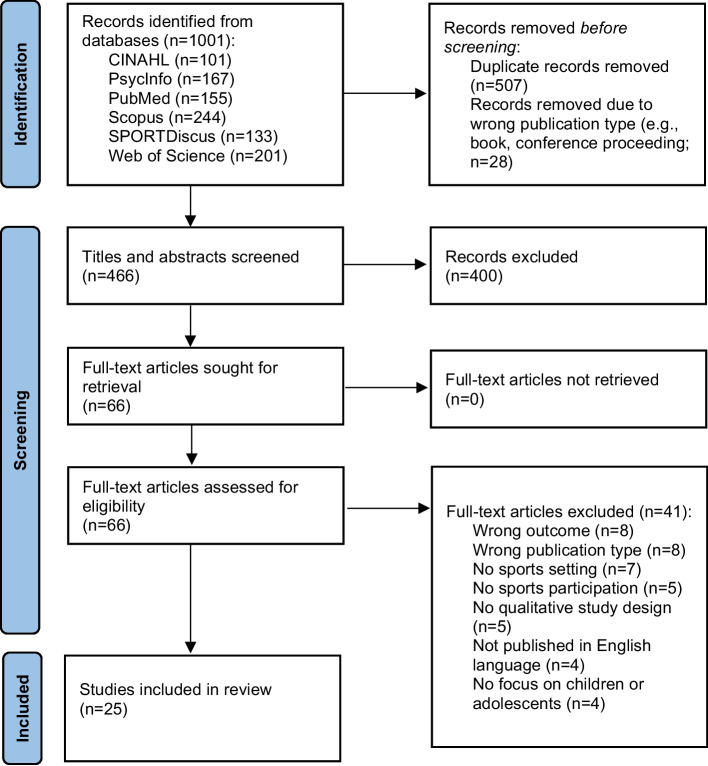
PRISMA flow diagram of study selection process

From the 1.001 records identified in the initial searches, a total of 25 studies were eligible for inclusion [33, 38, 39, 52–73] (see Fig. 1 for the PRISMA flow diagram of included studies). Table 1 provides a summary of the characteristics of the included studies. Studies were published between 2004 and 2024, with all but one published after 2012. Nearly half of the studies were conducted in Australia (*n* = 12; 48%). The remaining studies took place in the United Kingdom (*n* = 4), Sweden (*n* = 2), New Zealand (*n* = 2), Canada (*n* = 1), Finland (*n* = 1), Ireland (*n* = 1), and South Africa (*n* = 1). One study was conducted across multiple countries (i.e., in Argentina, Columbia, and Peru). Most studies (*n* = 23; 92%) used a cross-sectional qualitative design, while two employed a longitudinal qualitative approach. Eight studies (32%) specified a particular qualitative methodology: grounded theory (*n* = 3), case study (*n* = 1), phenomenology (*n* = 2), and ethnography combined with a (multiple) case study design (*n* = 2). Individual interviews were the most common data collection method (*n* = 15; 60%), and thematic analysis was the predominant analytical approach (*n* = 19; 76%). Only six studies (24%) provided a definition of mental health, primarily referencing the WHO (*n* = 4) [74, 81, 85, 89]. Similarly, six studies used a theoretical framework for data collection and/or analysis. These frameworks varied and included examples such as Tiatia-Seath’s Vai Niu framework [84], a positive psychology framework [87], and the psychosocial conceptual framework [88].

**Table 1:** Characteristics of the included studies

Author(s) (year of study) (reference)	Country	Aim(s)	Study design	Method of data collection	Method of data analysis	Definition of mental health	Theoretical framework	Participant characteristics	Sport discipline/sports participation level	Sports setting
Appelqvist-Schmidlechner et al. (2023) [52]	Finland	1) Investigate the mental health benefits of participation in organized sports in children participating in a sport-based PYD (Positive Youth Development) program targeted at socially vulnerable children and adolescents; 2) identify the underlying mechanisms contributing to these benefits; 3) explore the potential challenges and pitfalls in using sports-based programs for vulnerable children from the perspective of parents of participating children	Cross-sectional	Focus groups; semi-structured	Inductive thematic analysis	Yes, from WHO [74]	No	Parents of children (boys) participating in the Icehearts program (*n* = 28; 7 males and 21 females)	A variety of after-school team sports/grassroots level	Community setting: a sports-based positive youth development program
Brown et al. (2017) [53]	Australia	Explore the opinions and attitudes of parents of adolescent males (aged 12–17 years) who play sports regarding sports coaches as a gatekeeper for their son’s mental health	Cross-sectional	Individual semi-structured face-to-face interviews	Thematic analysis	No	No	Parents of adolescent males (aged 12–17 years) who were currently playing sport (*n* = 22; 8 males and 14 females)	Basketball, rugby league, soccer, swimming, tennis/not described	Sports clubs
Caperchione et al. (2022) [54]	Australia	Gain a deeper understanding of the perceived motivations and perspectives of participation in a mass participation event, such as the Sanitarium Weet-Bix Kids TRYathlon (SWKT) series, on children’s physical activity behavior, and overall health and well-being	Cross-sectional	Focus groups; semi-structured	Deductive thematic analysis	No	No	27 family units who participated in the SWKT event: children (*n* = 61; age range: 7–15 years; 26 males, 34 females, and 1 unknown) and parents (*n* = 31; 10 males and 21 females)	Triathlon/grassroots level	Community setting: the SWKT is a series of events held across Australia and New Zealand aimed at encouraging children and adolescents, aged 7–15 years, to get active and develop self-esteem in a positive social environment
Coyle et al. (2017) [55]	United Kingdom	Explore what mental health and mental health related behaviors mean for a group of young, elite athletes as conditioned by their peculiar social context as elite athletes	Cross-sectional	Individual semi-structured face-to-face interviews	Inductive thematic analysis	No	No	Elite divers (*n* = 8; age range: 14–24 years; 2 males and 6 females)	Diving/elite level	Elite diving club/squad
Drummond et al. (2022) [56]	Australia	Explore the views of young males and key stakeholders within sporting club settings to better understand young males’ mental health and well-being needs	Cross-sectional	Individual telephone interviews	Thematic analysis	No	No	Two young male athletes, two fathers and one mother (*n* = 5; mean age: 39.20 years; age range: 18–58 years)	Australian football, cricket/grassroots level	Two community sports clubs
Elliott et al. (2024) [38]	Australia	Explore the perceptions of various club stakeholders in relation to youth sporting clubs as spaces for mental health awareness, education, and training in the future	Cross-sectional	Individual semi-structured telephone interviews	Reflexive thematic analysis	Yes, but no specific reference	No	26 participants involved in community-level, organized youth sport: youth athletes (*n* = 9; age range: 12–17 years; 2 males and 7 females), parents (*n* = 8; age range: 37–58 years; 1 male and 7 females), and club leaders/coaches (*n* = 9; age range: 37–74 years; 6 males and 3 females). All participants were unknown to each other.	Australian football, cricket, netball, soccer, surf lifesaving, swimming, tennis/grassroots level	Community-level youth sports clubs
Ferguson et al. (2019) [57]	Australia	Gain a more detailed and nuanced understanding of how coaches perceive their role in the promotion of mental health for adolescent athletes and to understand their preferences for the content and delivery of mental health training	Cross-sectional	Focus groups; semi-structured	Thematic analysis	No	No	Coaches involved in coaching adolescents, mainly males, but also females (*n* = 20; mean age: 37.5 years; age range: 24–54 years; 18 males and 2 females)	Australian Rules Football, basketball, cricket, swimming, tennis/grassroots level	Local community-based sports clubs
Gervis & Dunn (2004) [58]	United Kingdom	Conduct preliminary investigations into emotional abuse in the coaching context	Cross-sectional retrospective	Individual semi-structured face-to-face interviews	Not described	No	Framework of emotionally abusive behaviors [75]	Former elite child athletes (i.e., at 8–16 years of age) (*n* = 12; mean age: 22.9 years; SD: 0.9 years; 4 males and 8 females)	Athletics, diving, gymnastics, hockey, netball, soccer/elite level	Elite sports setting
Gotfredsen et al. (2020) [59]	Sweden	Analyze how girls collectively construct responses to everyday stressors within the context of leisure participation, using a multiple case study based in northern Sweden	Longitudinal; ethnographic, multiple case-study approach	Participatory observations, repeated photo-elicited focus group discussions	Inductive thematic analysis	No	Stress process model [76], social practice theory [77]	Girls who were active members in two sport organizations (equestrian club and team ball game) in northern Sweden (*n* = 16; age range: 14–21 years)	Horse riding, team sport (ballgame)/grassroots level	Two sports organizations: one non-competitive and individual-based, and the other a competitive team-based sports club
Gulliver et al. (2012) [60]	Australia	Examine the perceived barriers and facilitators to help-seeking for mental health problems in young elite athletes	Cross-sectional	Focus groups; structured and open questions	Thematic analysis using both a priori and grounded codes; the ranked barriers and facilitators were quantitatively analyzed	No	No, but predefined list of barriers and facilitators for mental health seeking from the literature [78, 79]	Elite athletes (*n* = 15; mean age: 19.3 years; age range: 16–23 years; 9 males and 6 females)	Individual and team-based sports/elite level	The Australian Institute of Sport, which is the national sports training institute offering highly competitive scholarships to the most talented young elite athletes from around Australia. Athletes are provided with a wide range of facilities and services.
Hurley et al. (2017) [39]	Australia	Investigate 1) parents’ knowledge, attitudes and beliefs about adolescent mental health (i.e., their mental health literacy); 2) parents’ perceptions of the potential role of sport in mental health promotion and disorder prevention; 3) parents’ perceptions of the factors that might promote or limit participation in an adolescent mental health intervention delivered through community sport	Cross-sectional	Focus groups; semi-structured	Inductive thematic analysis	No	No	Parents of adolescent male athletes (*n* = 46; mean age: 46.3 years; age range: 31–63 years; 14 males and 32 females)	Australian Football League, basketball, cricket, soccer, swimming, tennis/grassroots and elite level	A combination of community and elite sports clubs
Lebrun et al. (2020) [61]	United Kingdom	1) Investigate talent development (TD) coaches’ experience of working with young elite athletes suffering from mental health issues (MHIs); 2) understand the needs of TD coaches and TD environments in order to best support TD coaches and their young performers	Cross-sectional	Individual semi-structured face-to-face interviews	Inductive thematic content analysis	No, but definition of mental health issues [80]	No	Coaches working in TD environments (*n* = 11; mean age: 42.55 years; age range: 30–62 years; 9 males and 2 females)	Athletics, judo, hockey, rugby, soccer, swimming/elite level (talent development)	Talent development sports environment
Marsters & Tiatia-Seath (2019) [62]	New Zealand	Address 1) how young Pacific male rugby union and rugby league players define mental well-being; 2) what young Pacific male rugby union and rugby league players’ perceptions and experiences are of pleasant emotions and mental well-being	Cross-sectional	Individual semi-structured face-to-face interviews, using the Pacific research methodological approach Talanoa	Grounded theory	Yes, from WHO [81], Galderisi et al. [82] and Keyes [83]	No	Young Pacific male rugby union and rugby league players (*n* = 20; mean age: 19.5 years; age range: 16–24 years)	Rugby/elite level	Pacific Rugby Union and Rugby League
Marsters et al. (2020) [63]	New Zealand	1) Identify and explore the protective factors for mental well-being among young Pacific male rugby union and rugby league players; 2) provide insight for future research foci to advance knowledge around Pacific mental well-being and suicide prevention for young Pacific male athletes	Cross-sectional; Grounded theory	Individual semi-structured face-to-face interviews, using the Pacific research methodological approach Talanoa	Grounded theory	No	Tiatia-Seath’s Vai Niu framework [84]	Young Pacific male rugby union and rugby league players (*n* = 20; mean age: 19.5 years; age range: 16–24 years)	Rugby/elite level	Pacific Rugby Union and Rugby League
Mazzer & Rickwood (2015) [64]	Australia	Examine what coaches perceive their role to be in supporting young people’s mental health	Cross-sectional	Individual semi-structured face-to-face interviews	Inductive and deductive thematic analysis	Yes, from WHO [85] and definition of mental health problems [86]	No	Community sports coaches of young people aged 12–18 years (*n* = 13; mean age: 36.1 years; age range: 23–59 years; 8 males and 5 females)	A range ​of community-level sports, including Australian ​Football League, basketball, cricket, netball, soccer, touch football, rugby league, rugby union and tennis/grassroots and elite level	A combination of community and elite sports clubs
Morrongiello et al. (2024) [65]	Canada	1) Explore youths’ perceptions of how peers and parents influence their skateboarding; 2) identify factors that affect their decisions about returning to the sport after injury	Cross-sectional; phenomenology	Individual semi-structured online interviews	Reflexive thematic analysis	No	No	Active recreational youth skateboarders who had experienced a medically treated injury while skateboarding during the past year (*n* = 26; mean age: 16.1 years; age range: 14–17 years; 87% male)	Skateboarding/grassroots level	Community setting: a recreational skateboarding community
Moss et al. (2020) [66]	United Kingdom	1) Ascertain the members’ perspectives and experiences of the community wheelchair basketball club; 2) identify the impact it had on their daily lives	Cross-sectional; phenomenology	Individual semi-structured face-to-face interviews	Inductive thematic analysis	No	No	Disabled and able-bodied members of a community-based wheel-chair basketball club: children (*n* = 9, age range: 6–16 years; 6 males and 3 females; 6 disabled and 3 able bodied); parents (*n* = 2; age range: 21–25 years; 1 male and 1 female; 2 disabled). Disabilities included: cerebral palsy (*n* = 4), postural tachycardia syndrome, spina bifida, hemivertebra and Duchenne’s muscular dystrophy.	Wheelchair basketball/grassroots level	A community-based wheelchair basketball club
Murphy et al. (2022) [67]	Ireland	Examine how physical activity holds different associations with perceived well-being in different contexts. More specifically, identify which aspects of leisure-time physical activity best support well-being.	Cross-sectional	Focus groups; semi-structured	Inductive thematic analysis	No	No	Post-primary students from five schools in the southeast of Ireland (*n* = 58; age range: 16–18 years; 27 males and 31 females)	A range of physical activities and sports, including athletics, basketball, dance, Gaelic/ladies football, gymnastics, hurling/camogie, rowing, rugby, and soccer/not described	Sports participation in different sports settings (community, sports clubs)
Page & Coetzee (2021) [68]	South Africa	Identify and document the perceptions of adolescents with spina bifida myelomeningocele and their primary caregivers on the factors that contribute to and hinder the well-being of adolescents living with spina bifida myelomeningocele in South Africa	Cross-sectional	Individual semi-structured face-to-face interviews	Inductive thematic analysis	No	Positive psychology framework [87]	Adolescents with spina bifida myelomeningocele (*n* = 7; mean age: 14.5 years; age range: 13–16 years; 5 males and 2 females) and their primary caregivers (*n* = 7; mean age: 49.5 years; age range: 39–75 years; 2 males and 5 females). The adolescent participants had varying degrees of ambulation.	Sport in general (e.g., volleyball, wheelchair dance)/grassroots level	Community setting: the Cape Flats, an expansive low-lying area southeast of central Cape Town, South Africa
Pittaway & Dantas (2022) [69]	Australia	1) Examine the impact of sport on acculturation and settlement experiences of South Sudanese resettled refugee youth in Australia; 2) explore young Sudanese encounters with racism and examine the potential of positive mental health outcomes associated with engaging in sports and sporting activity	Cross-sectional; case study	Individual semi-structured interviews; focus group discussions; community forums; literature review; analyses of newspaper articles	Thematic analysis	No	Psychosocial conceptual framework [88]	South Sudanese resettled refugee youth (*n* = 23; age range: 14–21 years; 16 males and 7 females); South Sudanese elders and parents (*n* = 11); local youth worker (*n* = 1); two community forums with stakeholders of the Sudanese community, elders, youth, social workers and parents (*n* = 65 and *n* = 47)	Basketball, soccer/grassroots level	Community setting: a local community outreach center in the southeast suburbs of Melbourne
Swann et al. (2018) [33]	Australia	Understand adolescent males': 1) knowledge and experience of mental health; 2) perceptions of organized youth sport as a vehicle for supporting mental health; 3) preferences/perceptions regarding needs and considerations for mental health interventions in organized youth sport	Cross-sectional	Focus groups; semi-structured	Inductive thematic analysis	Yes, from WHO [89] and Keyes [90]	No	Adolescent males who were currently playing in organized sports competitions (*n* = 55; mean age: 14.73 years; age range: 12–17 years); the majority participated at grassroots/local level, while a small minority also participated at regional/state level (i.e., for representative teams)	Australian Rules Football, basketball, cricket, soccer, swimming, tennis/grassroots and elite level	Community sports clubs; a small minority of the participants also participated at regional/state level (i.e., for representative teams)
Toyama et al. (2022) [70]	Argentina, Columbia, Peru	Explore the participants’ perceptions of which resources and activities help young people in large cities in Latin America to recover from mental distress in the form of depression and/or anxiety	Cross-sectional; Grounded theory	Online focus groups and online group conversations embedded into arts workshops; both semi-structured	Inductive content analysis	No	No	Adolescents (*n* = 65; age range: 15–16 years old), young adults (*n* = 80; age range: 20–24 years old) and professionals (*n* = 40; older than 18 years of age) that had experience of professionally working with young people (psychologists, teachers, youth workers, psychiatrists, other). Of the young people, 69% have previous experience of anxiety and/or depression. And of these, 55% have previous experiences with mental health treatment.	Sports activities in general/grassroots level	Community setting: the study was conducted in the capital cities of three Latin American countries (Argentina, Colombia, and Peru), and focused on young people from poorer economic neighborhoods based on national indicators of income
Vaughan et al. (2022) [71]	Sweden	Examine how the social and cultural seriousness associated with football acts as a constraint on player development, cascading into athlete development environments, and influencing players’ psychological well-being and skill development	Longitudinal; 18-month ethnographic case study	Participant observations; document analysis	Phronetic iterative qualitative data analysis	No	Ecological Dynamics rationale [91, 92], conceptual Skilled Intentionality Framework [93, 94], Athlete Talent Development Environment [95]	N.A.; children (boys), parents and coaches at a professional soccer club are observed	Soccer/elite level (including talent development)	AIK football club: a professional soccer club and academy
White & Bennie (2015) [72]	Australia	Investigate gymnast and coach perceptions about the development of resilience through youth sport	Cross-sectional; Grounded theory	Individual semi-structured face-to-face interviews	Inductive qualitative data analysis	Yes, from Australian Institute of Health and Welfare [96]	No	Female gymnasts currently participating in gymnastics training and competition with gymnastics’ level 2–7 (*n* = 22; mean age: 12.5 years; age range: 10–16 years) and gymnastics coaches coaching at gymnastics’ levels 1–7 (*n* = 7; mean age: 22.14 years; age range: 18–30 years)	Gymnastics/grassroots level	Two community gymnastics clubs
Williams et al. (2013) [73]	Australia	1) Reveal why young people who had experienced psychosis consistently decided to attend the street soccer group; 2) explore participants’ perspective of their experience of the group	Cross-sectional	Individual semi-structured face-to-face interviews	Thematic analysis	No	No	Patients using the early psychosis tertiary mental health service who were regularly attending the Street Soccer program (*n* = 6, age range: 18–23 years; all males). Four participants had a diagnosis of paranoid schizophrenia, one psychosis not otherwise specified and one bipolar affective disorder.	Soccer/grassroots level	Community setting: the Street Soccer program is a once-weekly group program that is run in a local community park. The group session is open to all young people living on the Gold Coast but is targeted towards young people experiencing difficulties such as homelessness, mental illness or drug and alcohol issues.

Ten studies focused on adolescents as the target population. Seven studies focused specifically on adolescent males, and four focused on both children and adolescents. The remaining studies focused on boys (*n* = 2), adolescent females (*n* = 1), and girls and adolescent females (*n* = 1).

In five studies, research participants were adolescents. Four studies included only adolescent males, and one included only adolescent females. Three studies included only coaches, and three included only parents. Eight studies involved mixed participant groups—such as children, adolescents, parents, coaches, and other stakeholders—often of both genders. Of these eight studies, three specifically included children (both genders = 2; girls = 1) as participants. One study did not directly involve participants, relying mostly on researcher observations. Total participant numbers ranged from 5 to 185 individuals per study.

Studies focused on both individual and team sports (*n* = 11), team sports only (*n* = 8), or individual sports only (*n* = 4). Two studies referred to sports activities in general without specifying the type. Across studies, different sports were practiced, such as athletics, Australian football, basketball, diving, gymnastics, and tennis. In 13 studies, sport was primarily practiced at a grassroots level with some competitive elements. Seven studies focused exclusively on elite-level participation, three included both grassroots and elite levels, and two did not clearly specify the level of participation. Only four studies reported on the frequency and duration of sports participation [55, 59, 72, 73] (not included in Table). Sports settings varied across the studies and included community sports clubs (*n* = 7), professional or elite sports clubs (*n* = 7), a combination of community and elite sports clubs (*n* = 3), and community settings outside organized sports (*n* = 7). One study involved multiple sports settings.

### Quality of studies

The overall quality of studies was good. Using the 10-item CASP checklist, 21 studies (84%) met seven or more criteria, indicating that the findings of this review are reliable. All or nearly all studies scored positively (‘yes’) on clearly stated aims, appropriate qualitative design, data collection and analysis methods, ethical considerations, clarity of findings, and value of results. However, for most studies (*n* = 24; 96%), it was unclear (‘can’t tell’) whether the recruitment strategy was appropriate, due to limited descriptions of participant selection and non-participation reasons. Additionally, only five studies (20%) adequately addressed the impact of the researcher-participant relationship, while most (*n* = 15; 60%) provided no discussion of this aspect. For full details of quality assessment of each study, see Additional file 3.

### Results of the thematic analyses

The results of the thematic analyses are presented in Table 2. The results are presented according to the three main topics: 1) Changes in mental health outcomes (intrapersonal level of the socio-ecological model): 2) Individual mechanisms (also intrapersonal level of the socio-ecological model); and 3) Factors in the sports setting (interpersonal level, and organizational and community levels of the socio-ecological model). The main themes and their corresponding sub-themes (with sub-themes presented in italics in the text) are explained in more detail below. In general, main themes applied to both children and adolescents, boys and girls, grassroots and elite sports participants, and sports participation inside and outside sports clubs. Only minor nuances in differences were observed at the sub-themes level. When results apply to a specific subgroup, this is described accordingly. Moreover, positive experiences of sports participation on mental health and facilitating factors in the sports setting were more often reported than negative experiences or hindering factors. Author comments and participant quotes (presented in italics and enclosed in quotation marks) are included in the text to illustrate the themes. Please see Table 2 for an overview of themes, sub-themes as well as examples of the authors’ interpretations and illustrative quotes. Additional file 4 presents an overview of themes, sub-themes and accompanying references.

**Table 2:** Results of the thematic analyses

Main theme*	Sub-theme* (specific subgroup)**	Example of author interpretation or participant quote
**Changes in mental health outcomes**		
***Intrapersonal level—Child or adolescent***		
*Emotions*	Positive emotions through sports participation (+)	Furthermore, Jessica and Sophie identified that the effects of playing wheelchair basketball were not merely transient and benefitted their mental health by making them happy or providing an escape from the stresses of daily life: ‘You stay happy, maybe for the rest of the week cause you’ve had fun and remembered that feeling.’ (Author comment/sports participant quote, p.123) [66]
	Negative emotions through sports participation (-)	The participants felt that feelings of depression and anxiety could result from poor performance: ‘I think after, if maybe after a competition you don’t perform very good, you can get depressed and stuff.’ (Author comment/sports participant quote, p.4) [60]
	Learning to regulate negative emotions (+) (G)	Several participants discussed the mental health benefits. One commented: ‘I love skateboarding […] even if I get injured, that love really motivates me. It helps me emotionally. It is worth any pain that it brings when I fall.’ (Author comment/sports participant quote, p.5) [65]
*Self-confidence and self-esteem*	Increased self-confidence or self-esteem (+)	‘After her swimming lesson I said do you want to practice so we can build that stamina up she was more than happy and willing and then when she did finish it she was very proud of herself. So that definitely helped boost her confidence and you know, driver for her to engage in things that she’s not familiar with.’ (Parent quote, p.6) [54]
	Decreased self-confidence or self-esteem (-)	Third, a couple of the parents expressed their concern of exposing their children to ongoing failures. Icehearts teams also compete against regular sports clubs with greater focus on sports performance. Some of the parents were afraid that they put their children at risk of mainly experiencing failures and lost games when competing against these clubs. They were afraid that these experiences would damage the already fragile self-esteem of their children. (Author comment, p.252–253) [52]
*Stress*	Relaxation (+)	Others considered sport to be ‘a happy place’ where they can relax and get away from problems: […] ‘Whenever I'm feeling stress I'll [go to a basketball court] and shoot, just because it's a happy place for me. It feels like a sense of home. As soon as you find a sense of home, that's where a lot of people should be able to go … wherever you find yourself most comfortable, that's where you should try and go, try and clear your mind and trying to sort things out.’ (Author comment/sports participant quote, p.59) [33]
	Stress through sports participation (-)	‘Playing sport, especially like cricket, it can get you down a lot. If you're not scoring runs or playing well, you can get a bit stressed.’ (Sports participant quote, p.59) [33]
*Resilience*	Learning to handle setbacks (+) (G)	Learning to deal with or respond to losses and setbacks was highlighted by the majority of the team and individual sport participants in contributing to progressions over time and in developing resilience: ‘Yes, you have to learn the skills to deal with loss because you’re going to experience so much failure and setbacks in life. You have to learn to deal with it. And that’s a good thing in doing sports: it teaches you that you’re going to have setbacks. Then at least you’re prepped. If the first time you experience a setback is in school, you fail a test, you’re going to be completely doubting your abilities, whereas you learn that from a young age.’ (Author comment/participant quote, p.8) [67]
**Individual mechanisms**		
***Intrapersonal level—Child or adolescent***		
*Distraction and focus (* +*)*		‘I’m so concentrated on the game, I don’t really think about other problems going on in my life…so I guess it’s a good distraction…it definitely positively impacts my mental health, because I’ve suffered with depression…so that’s kind of what pushed me to get involved.’ (Sports participant quote, p.123) [66]
*Performance*	Performing well (+) (A)	‘[Positive mental wellbeing] is when I’m playing well. And like we have skinfold tests and fitness tests and if my skinfold is going down then I feel real happy and feel like I’m in the best shape to play.’ (Sports participant quote, p.10) [62]
	Feeling pressure to perform (-)	Such focus on performance was reported to involve greater exposure to factors such as stress and pressure, and was therefore more likely to be linked with negative influences on mental health. (Author comment, p.60) [33]
	Unsuccessful performance and (fear of) injury (-)	The centrality of sports for participants’ identity and mental wellbeing meant that some participants experienced significant psychological distress when their performance declined or their athletic identity was challenged: ‘I’ve been pretty injury prone. It was stressful not being able to play and you start to get depressed at that. Especially when I was still at school, when I was focused on nothing else but rugby. I just stopped going to school and it was a depressing time not being able to play and then you get back do your rehab and play…and then another injury and you just feel like giving up.’ (Author comment/sports participant quote, p.68) [63]
*Personal development*	Improving oneself (+) (A)	Each participant was asked what the most enjoyable aspect of engaging in physical activity and sport was, with improvements over time being the most common response: ‘I think definitely the chance of getting better at things. I used to hate running but I got into it and I started to like the sense of achievement after finishing it and to notice that you could get faster or you could run a bit longer and you were starting to find it easier, as you were getting fitter and getting better at it. I just really liked that.’ (Author comment/participant quote, p.4) [67]
	Physical competence in sports (+) (G)	Besides skills through competitive sports, perceived physical competence also contributed to mental health benefits observed by the parents. Increased self-esteem and self-confidence were the most reported mental health benefits, commonly resulting from increased competence in sports and from experiences of succeeding. (Author comment, p.250) [52]
	Experiences of succeeding (+) (G)	Parents described how their child took pride in completing the race and looked forward to future races, and a number of parents noted how competing in the TRYathlon bolstered their child’s confidence and resilience. These feelings were subsequently met with feelings of happiness and relief post-event. (Author comment, p.6) [54]
	Learning life skills through sports (+)	Furthermore, the parents reported that the children had learned to take responsibility and to follow rules, which has resulted, for instance, in increased self-esteem. ‘And then he has learned to take responsibility, responsibility in his team. And that has improved his self-esteem… At some point he was some kind of captain of his team, and I think this was done on purpose. The mentor wanted to give him responsibility so that he also learns to pay attention to other people.’ (Author comment/parent quote, p.250) [52]
*Purpose and structure in life*	Purpose or meaning (+)	Diving also helped participants stay, by their own definitions, mentally healthy. For example, having a sense of direction, purpose, self-discipline and motivation. For Ester, diving enabled ‘you get yourself out of bed in the morning and do your exercises and like when you're younger more discipline.’ Similarly, Mike said diving ‘motivates to do something in the day, I think my mental health is a lot better that I do diving.’ (Author comment/sports participants quotes, p.13) [55]
	Providing structure (+) (A, G)	‘I think they just go hand in hand together. Without sport, I wouldn’t be the same person I am, definitely, without sport. I wouldn’t be as happy as I am, or as much friends as I have, and all that. It gives me something to do, wake up every day, and go out, and know that I am doing something with myself. If I didn’t have sport, I would just be sitting around doing nothing. I’d be wasting …’ (Sports participant quote, p.5–6) [67]
*Sense of belonging (* +*)*		The themes mentioned by youth demonstrate the significant influences that peers have on their skateboarding. Engagement with peers promotes their psychological well-being and creates opportunities to build and deepen relationships. It is an activity that creates a deep sense of feeling supported and belonging to part of a larger valued community. (Author comment, p.4) [65]
**Factors in the sports setting**		
***Interpersonal level—Coach***		
*Coaching style*	Encouragement (+)	These responses highlighted how the overall style preferred by parents was a gentle, positive and encouraging one. (Author comment, p.7) [53]
	Positive (constructive) feedback (+) (G)	Through the positive coaching approach and pedagogical skills, for example through positive feedback, the parents had noticed a remarkable increase in the self-esteem and self-confidence of the children. ‘The self-confidence of X has increased hugely. He started to believe in himself and in what he is doing when somebody praised him as he succeeded in something. These experiences were then also transferred to school and to other fields of life.’ (Parent quote, p.251) [52]
	A positive attitude (towards challenges) (+) (G)	‘They teach me that… if you think you can do something you probably can but if you fill your mind with negative thoughts and you think you can’t then you won’t be able to. So, they sort of teach me how to believe that I can do something.’ (Sports participant quote, p.387) [72]
	Creating a welcoming, safe and fun learning environment (+)	‘Mainly because it’s just got a real lighthearted feel to it and it’s just fun to be able to kick a ball around and not have that sort of pressure of a real game.’ ‘He [the coach] is very welcoming and open to who you are.’ (Sports participants quotes, p.608–609) [73]
	Too much focus on competition or performance (-)	‘It depends on the people you’re around. For example, I do my football and it’s really good for your wellbeing but then you have your coaches and they can really put you down…They made everyone so competitive where we were all against each other on the team when we were supposed to be playing together.’ (Sports participant quote, p.7) [67]
*Coach-child relationship*	Strong emotional and trusting bond (+)	The relationship/rapport that the coach has with the young person was a strong sub-theme overall. Participants [parents] mentioned this theme in a variety of ways. For example, if the coach and player/youth have a strong existing relationship, trust and open conversation are more likely. It may also allow the coach to feel more comfortable that they can support and help the youth (Author comment, p.8) [53]
	Noticing (behavior) change (+)	‘I think it would be through observation, through communication with the player. I’d suggest that in most cases it is observation, it is probably a tacit thing of having seen a very large number of boys, young men at that age. And also knowing that individual and knowing what their average behaviour is. And seeing how their body language and seeing how their training behaviour, how their behaviour generally changes.’ (Coach quote, p.46) [61]
	Open communication (+)	Participants perceived positive coach-athlete relationships to be built upon open and honest communication between gymnast and coach. These relations were also beneficial if the coach was approachable and trustworthy: ‘When they tell me about their day and I tell them about mine, it’s building a better relationship and more trust.’ Robust relationships meant gymnasts were comfortable asking their coach questions and confiding in them when they faced problems inside and outside the gym. This reassured gymnasts that their coach would provide emotional support during difficult times. (Author comment/coach quote, p.386) [72]
	Emotional support (+)	Alana made particular mention of her coaches in supporting her with the challenges associated with depression: ‘At first I don't think [coaches name] had ever had anybody like that, and he was very good. He used to say they're called gremlins and he used to like, when I was little to kind of like, not focus on them, tell them to go away, or whatever. And, he still knows about it now and to be honest he knows that I wouldn't do it on purpose, so, I wouldn't not just because I don't want to do the dive, to have that trust.’ (Sports participant quote, p.14) [55]
	Lack of trust and open communication (-)	This decision [to talk to the coach about (mental health) problems] appeared to depend on their relationship with the coach. Some adolescents stated that trusting the coach was important. Others, though, suggested that they would not talk to their coach unless they felt like the coach knew more about mental health, or felt that they could take the coach more seriously. (Author comment, p.60) [33]
***Interpersonal level—Teammates and peers***		
*Inter-peer relations*	Positive social relationships/friendships (+)	Youth talked about the ‘community’ that skating offered and many commented on how important this was to keep them skating. Skateboarding was associated with a deep sense of belongingness with peers: ‘[…] skateboarding brings us all together.’; ‘You make so many friends […] and become a big community and it’s special.’; ‘[…] I’ve made a lot of really great friends. There’s like this instant connection from skating and this keeps me skating […].’; ‘There’s so much of a community there and you get to know everyone somehow. It’s like a family but of friends.’ (Author comment/sports participants quotes, p.4) [65]
	Shared experiences (+) (G)	Sharing the experience of winning, losing and progressing over time was said to be more enjoyable than on your own: ‘The best thing is winning but celebrating it as a team together. We have that one big thing in common and can share the experience, especially after the few months of working together.’ (Author comment/sports participant quote, p.8–9) [67]
	Support (+)	‘I think the brotherhood is the best part about playing rugby. Just meeting new people and complete strangers and then by the end of the year it’s like you’ve grown up with them. When you go on the field it’s that mana (spiritual power) inside you that you link to your other brothers. Brotherhood is a big part of playing the game. Everyone’s just amping each other up and got each other’s back, like that mentality if one’s down then the rest of the pack is there to pick you up.’(Sports participant quote, p.64) [63]
***Interpersonal level—Family***		
*Family-child relationship*	Support (+) (A)	These participants reflected on the importance of family in relation to supporting mental health through sport: ‘Family is … there to guide and support you through everything you go through, especially parents because they've gone through it themselves. They've had ups and downs in their life. What they see you going through they can recognise and help push you through it, and if they can't see it, it's good to go talk to them because then they'll probably know ways to help you get through things and find answers to get better or help others … I think the good thing about playing basketball is that we go on a lot of road trips, so the family comes together in the car.’ (Author comment/sports participant quote, p.60) [33]
	Focus on competition/performance (-)	The coaches also suggested that parental pressure and their focus on results and winning could be a cause of anxiety for athletes. One coach noted that the parents sometimes play a role in driving kids away from sport: ‘The parents, to a certain degree, drive them away because they see their parents telling everyone how good their kids are, and the kid’s getting all this pressure.’ They highlighted that the car journey home could be a particular time that is stressful for athletes and that parents can at times make this situation worse by wanting to talk about results when the emotional response is still raw for the athlete: ‘As a parent you’re going to be disappointed, and you’ve got to take the emotion out of the situation.’ (Author comment; coaches quotes, p.245) [57]
***Interpersonal level—Other (team) professionals***		
*Professional-child relationship*	Support (+) (E)	‘[…] And then I had a like, [psychologist name], she was brilliant. And I don't know what she said but she just used to talk to me and it used to kind of just blow things away. And we've got a new one in now and she's lovely and we're working on things but I heard from [psychologist name] this year she doesn't work for us anymore and I just had one conversation with her and then like, unfortunately it doesn't work like that anymore. I definitely want to keep in contact with her.' (Sports participant quote, p.15) [55]
	Lack of trust or support (-) (A, E)	‘I probably would have been a bit more open to talk … but because we did have staff that were there to help, but it felt like they were so far away even though they were in the group of the coaches. Like there were psychologists and that kind of feeling… [but] that was a bit daunting, [but]… if it was coaching staff or someone it would probably be easier to open up.’ (Sports participant quote, p.449) [38]
***Organizational and community levels—Social climate***		
*Social climate at the sports club/location*	Challenging, safe and nurturing environment (+) (G)	‘He definitely feels safe and I’m lucky I’ve got kids that speak about their feelings and I’ve got one, my middle child suffers from anxiety and depression, so he feels safe around his sporting club. He uses it to deal with some of those issues. When I say anxiety, he uses it to sort of address it and because it’s a comfortable environment, he feels like he can push himself or let himself be – try different things and fail because he gets to repeat it again quickly and try it again, rather than have to sit on that moment it didn’t go so well for him for the next week, he gets to get up and go and try again.’ (Parent quote, p.6) [56]
	Too much focus on competition/performance (-)	I’m observing the 07 group (players born in 2007); the intensity is frantic as tackles fly in left, right, and center. However, these periods of ferocity are punctuated as players break into tears. More often than not the pain isn’t physical, but mistakes bring gasping sobs and injuries are feigned to cover over the emotional turmoil. I turn to Matthias and ask if this is normal, he nods…It’s hard not to be impressed by the speed and skill of the individual play, but it’s equally hard not to despair that kids of 10 years old are feigning injury and crying because they’ve made a meaningless mistake…It’s obviously not meaningless to them; again, I’m left wondering why? (Author fieldnote, p.10) [71]

^*^ + = positive mental health outcome or positive influence on mental health;-= negative mental health outcome or negative influence on mental health

^**^A adolescents, *G* grassroots sports, *E* elite sports

### Changes in mental health outcomes (intrapersonal level)

Mental health was experienced in different ways. This is evident by the ways mental health and changes in mental health through sports participation were described by individuals. Mostly, positive influences of sports participation on mental health were reported, but sometimes these influences were negative. Four main themes were identified: 1) Emotions; 2) Self-confidence and self-esteem; 3) Stress; and 4) Resilience.

#### Emotions

Children and adolescents experienced *positive emotions* through sports participation, in particular feelings of happiness, enjoyment, and pleasure, but also emotions such as pride, hope, accomplishment, and courage [33, 39, 52, 54, 55, 59, 62, 65–69, 72, 73]. Sometimes *negative emotions* were described, such as anxiety, depression, anger, feeling scared, and feeling upset [33, 55, 57, 58, 60, 63, 67, 71, 73]. For some children and adolescents, particularly those participating at the grassroots level, sports offered a way to *learn to regulate negative emotions* [52, 59, 65, 70, 72], as described by this mother about her child:

*‘Icehearts has been a team where you won’t be kicked out even if you shout to other kids. You are able to train and learn to control your emotions and he is doing quite well now. He can already handle disappointments… Hot-headed behaviour in the field has turned into a more reasonable playstyle, and he hasn’t lost his temper recently.’* (Parent quote, p.250) [52].

#### Self-confidence and self-esteem

Sports participation *increased feelings of self-confidence and self-esteem* [52, 54, 57, 63, 65, 67, 68, 72, 73]. This was mainly experienced in relation to successful performance and personal development (see also: individual mechanisms). However, in opposite situations, such as when there were failures in sports, this could *decrease a person’s self-confidence or self-esteem* [52, 53, 56, 58].

#### Stress

Children and adolescents reported experiencing stress, such as from school, family or peers. Participating in sports helped to *relax from these daily stressors* and to experience a sense of peace or calm [33, 52, 59, 66, 67, 69, 70, 72, 73]. However, sports participation itself could be an *additional source of stress*, for example, in competitive situations or for those with a greater focus on performance (see also: individual mechanisms) [33, 55, 59, 63, 72]. In this regard, balancing sports with other life-demands (e.g., school, friends) was also experienced as stressful for some [33, 59].

#### Resilience

Sports participation offered different challenges. Through competition, experiences in playing in a team, and learning new (difficult) skills, children and adolescents were provided with different situations to try something, fail, and try again until they succeeded. Particularly at the grassroots level, these experiences provided valuable opportunities to *learn how to handle setbacks*, not only in sports, but in life in general [52, 54, 57, 67, 69, 72]:

*‘When they’re doing a skill and they fall off the beam or the bars, it takes a lot of effort to get back up, to get over the fear and start again. You use that a lot out of gym as well … at school you do an exam, you fail, you have got to pick yourself back up and do it again, and I think that’s a really important skill that you learn here.’* (Coach quote, p.385) [72].

### Individual mechanisms (intrapersonal level)

There were different mechanisms reported on how sports participation influenced mental health. Five main themes were identified: 1) Distraction and focus; 2) Performance; 3) Personal development; 4) Purpose and structure in life; and 5) Sense of belonging.

#### Distraction and focus

Sports provided *distraction* from daily stressors, worries, negative thoughts, and negative emotions, because participation in sports required participants to direct their attention to something else, such as *focusing* on the game or performing a specific sports skill [33, 54, 56, 59, 63, 65–67, 69, 70, 72, 73]. Children and adolescents described this, for example, as ‘emptying your head’ [59] and an ‘escape from reality’ [65]. Also, flow-like states were described in which participants were fully immersed in the sports activity and nothing else mattered [33, 67, 69]:

*‘Basketball it keeps you from doing bad stuff. Yeah, keeps you focused. But sometimes it takes over, you forget about school, church and family and just play basketball. You get into it, nothing else matters.’* (Sports participant quote, p.7) [69].

Sometimes, this positive and relaxed feeling lasted after the sports activity and made it easier for children and adolescents to solve a specific problem or to concentrate on their homework [59, 66, 67].

#### Performance

Central to this theme is the performative nature of sports. Adolescents, in particular, reported experiencing positive mental health when they *performed well*, especially when they were at their best. For example, when they had optimal performances during training or competitions or when they were in optimal physical shape [33, 55, 62, 63, 67–69]. However, *unsuccessful performance*, especially in competitive situations, and *(fear of) injury* were associated with decreases in mental health among both children and adolescents [33, 39, 52, 55, 59, 60, 62, 63, 65, 67, 71, 72]. Also, too much focus on performance (i.e., whether self-imposed or driven by external sources; see factors in the sports setting) could enhance *internal pressure*. Consequently, some children and adolescents experienced increased stress and negative emotions through sports participation [33, 39, 53, 55, 57, 59, 60, 62, 63, 67, 71].

#### Personal development

Sports participation offered opportunities to improve oneself and to master new (sports) skills, which in turn enhanced self-confidence, self-esteem, and positive emotions. In this regard, adolescents emphasized the importance of *self-improvement* [33, 55, 57, 62, 67]. For participants at the grassroots level, *increased physical competence in sports* [52, 57, 65, 67] and the *experience of succeeding* (e.g., finishing a sports task or event) were especially important [52, 54, 65, 67, 72]. In addition, *learning other life skills* (e.g., taking responsibility, problem solving, goal setting, collaboration, and leadership) contributed to the mental health of both children and adolescents [33, 39, 52, 57, 67, 72].

#### Purpose and structure in life

Sports participation was reported to provide a sense of direction, motivation, and discipline, for example, by defining clear goals in sports (e.g., getting better, increasing fitness, building-up muscle, achieving something). In this way, children and adolescents experienced *purpose or meaning in life*, which helped them to stay mentally healthy [52, 54, 55, 66–69, 73]. Furthermore, for adolescents involved in grassroots sports, participation offered *structure* to their week through weekly recurring activities. It also helped them to stay focused on longer-term goals and to avoid destructive behaviors, such as the use of drugs [59, 67, 69, 73]:

*‘Sport is the answer, there are two sports that are dominant, basketball and soccer. Some join teams and work hard to build up muscles and fitness and cardio, so it makes them not to use drugs, because it avoids being a good athlete. I heard a lot talked of that and it actually draws them away from that behaviour.’ […]* (Sports participant quote, p.7) [69].

#### Sense of belonging

Social connectedness or *sense of belonging* is another important theme contributing to the mental health benefits of sports participation [52, 54, 56, 57, 59, 63, 65–67, 70, 72]. Sports are often done with teammates or peers. Being part of a team or community, doing something together, sharing the same interest and having a common purpose all contributed to a connection or sense of belonging, which was related to positive mental health outcomes, such as positive emotions, increased confidence, and less stress.

### Factors in the sports setting

From the perspective of the socio-ecological model, factors in the broader sports environment can influence the relationship between sports participation and mental health. Based on the findings of the current review, factors at the interpersonal level, such as interactions with the coach, teammates and peers, family members, and other team professionals appeared important. At the organizational and community levels, the broader social climate of sports participation influenced the mental health of children and adolescents. No important themes were identified regarding the policy level.

### Coach (interpersonal level)

Studies showed how the coach played a key role for the mental health of children and adolescents through sports participation. This could be both positive and negative. Two main themes were identified: 1) Coaching style; and 2) Coach-child relationship.

#### Coaching style

A positive coaching style contributed to the mental health benefits of sports participation, such as increased self-confidence or self-esteem. Positive coaching was experienced when the coach was *encouraging* [53, 57, 64, 72, 73]. For children and adolescents participating at the grassroots level, it was also important that coaches provided *positive (constructive) feedback* (e.g., focusing on things that go well, praising the child for its effort) [52, 56, 57, 72, 73] and maintained a *positive attitude towards challenges* rather than becoming stressed or frustrated [53, 57, 72]:

*‘If you go into the gym with a good attitude then you’re going to have a good day, but if you go in thinking that you can’t be bothered then you may as well not do it. She [the coach] always says positive thoughts lead to positive results.’* (Sports participant quote, p.387) [72].

This approach was perceived to help grassroots participants develop a resilient, ‘never give up’ attitude.

In addition, mental health of children and adolescents was enhanced when the coach *created a welcoming, safe and fun sports environment* [33, 52, 57, 64, 73]. This was described as a sports environment in which youth felt welcome (e.g., everyone can join), safe (i.e., making mistakes is ok; the feeling someone is looking after you), and there was a focus on the fun aspects of participation. On the contrary, when a coach *focused too much on competition or had (unrealistic) high expectations about performance*, this could enhance internal pressure among children and adolescents (see also: individual mechanisms) and consequently, negatively affect their mental health [33, 53, 58, 60, 67, 71]:

*‘I think some of the negatives is that you can get a coach who are quite aggressive and have high expectations of these boys, they’re still boys and if you try to treat them as FIFA world class players then that can put pressure on them.’* (Parent quote, p.7) [53].

#### Coach-child relationship

Most coaches can see children and adolescents on a weekly basis in an informal social setting, and often over an extended period of time (sometimes multiple years). Building *a strong emotional and trustworthy bond* between the coach and the young person, by spending much time together and paying attention to the individual, was the basis for mental health benefits of sports participation [33, 52, 53, 57, 64, 72]. When the coach and young person had a strong and trustworthy relationship, the coach was more likely to *notice any (behavioral) changes* related to mental health [53, 57, 60, 61, 64]. Strong trustworthy relationships also meant children and adolescents were more comfortable asking their coach questions, and confiding in them when they faced (mental health) problems inside and outside sports (i.e., *open communication*) [33, 38, 53, 55–57, 60, 61, 64, 72]. This provided reassurance, and the feeling of being supported and heard. It also offered the opportunity for coaches to provide direct *(emotional) support*, for example, by listening, letting know they cared and searching for potential solutions to a problem [33, 39, 52, 53, 55, 61, 64, 72]:

*‘We can tell them [the coaches] anything and they'll help us through it, everyone's really supportive.’* (Sports participant quote, p.14) [55].

Overall, these kinds of coach-child relationships appeared to support children and adolescents through both adversity and success (in sports) and enhanced their mental health.

However, sometimes there was *a lack of trust and open communication* [33, 38, 56, 57, 60, 61]. In these situations, children and adolescents were not always confident to share their negative (sports) experiences (e.g. poor performance, injury, pressure); sometimes, because they were afraid of negative consequences (e.g., not being selected for a team, being seen as weak). In addition, coaches were not always open about certain sports decisions they made (e.g., about team selection, content of a specific training). Honesty and transparency were important for children and adolescents to understand and interpret these decisions. In both situations, they did not feel understood and heard, which was reported to contribute negatively to their mental health.

### Teammates and peers (interpersonal level)

#### Inter-peer relations

Sports participation offered the opportunity to engage with peers with the same interests and to spend a lot of time together. In this way, children and adolescents were able to build and deepen *relationships*, which often resulted in (new) *friendships* and an enhanced sense of belonging (see also: individual mechanisms) [33, 52, 56, 57, 59, 63, 65–70, 72]. These relationships were also described as being part of a ‘community’ [52, 65], ‘(a second) family’ [56, 59] or ‘brotherhood’ [63]. For grassroots participants in particular, *sharing experiences* played a key role in protecting or enhancing mental health [59, 65, 67, 72]: sharing positive experiences, such as getting better or winning together enhanced the connection between peers and resulted in more enjoyment. Likewise, sharing negative experiences, such as stressors at home or poor performances and failures in sports, made it a shared problem or failure which protected their mental health. In addition, sporting peers at both grassroots and elite levels provided *support* in various ways, for example, in the form of encouragement to get better, by helping to learn a new sports skill, or by cheering one up or reflecting on difficulties, such when someone performed poorly [33, 39, 52, 53, 55, 56, 59, 60, 63, 65, 67, 70, 72]:

*‘At first, you’re just annoyed at yourself, but then I think with the people around you, they kind of tell you, “Ah, yeah, you can learn from that and move on.” It will help you going forward. So it’s kind of both.’* (Sports participant quote, p.8) [67].

This support network was also related to enhanced self-confidence. So, teammates and peers were very important in the relation between sports participation and mental health.

### Family (interpersonal level)

#### Family-child relationship

*Family support* was particularly important for adolescents, helping them to stay grounded, hopeful, and motivated to grow and improve in their sports [33, 39, 53, 55, 62, 63]. For elite sports participants, having family members who understood the pressures, commitments, and mental strain associated with playing elite sports, was a protective factor for their mental health [62]. Furthermore, the reassurance of ‘unconditional’ family support when adolescents experienced some sort of perceived failure was an important protective factor for their mental health [33, 62, 63]:

*‘Last year in the semi finals, I got concussed in the first 10 min. That was my second concussion in three weeks and it took me out of the game. I couldn’t help my team and we lost and that sort of took me out of contention for the secondary schools camp. The thing that kept me going was knowing that my family was there to support me and they kept telling me I had another year to make things right. They just kept reminding me that I’m still young and still a kid and I’ve got to have fun doing the things I do, I’ve got to enjoy yourself.’* (Sports participant quote, p.62) [63].

However, when family, in particular parents, were *too focused on results and winning*, this could enhance internal pressure (see also: individual mechanisms) and, consequently, stress and negative emotions among both children and adolescents [57, 60, 63, 71]. So, family could have both a positive and negative influence on the relationship between sports participation and mental health.

### Other (team) professionals (interpersonal level)

#### Professional-child relationship

In elite sports settings in particular, children and adolescents often had access to managers and/or specialist support staff, such as sport psychologists, lifestyle coaches, or nutritionists. However, children and adolescents had different experiences of this formal support in relation to their mental health. Some experienced *support* from these professionals regarding their mental health, because these were professionals they felt comfortable to talk to and ask for advice when they experienced (mental health) issues related to sports [38, 53, 55, 60, 61, 63]:

*‘Every two weeks we have a lifestyle coach and a psychologist who comes down and now that's all set up I think everyone is a lot happier because every two weeks they can vent their frustrations and issues with the psychologist so it's not building up within the group or within them so I think that is a good thing that has been put in place.’* (Sports participant quote, p.14–15) [55].

However, some adolescents were *not confident* in sharing their sport problems with these professionals or experienced *a lack of support*, because professionals focused too much on performance (and not on their mental health) and, sometimes, even exacerbated problems [38, 55, 60]. In this sense, other (team) professionals could both have a positive and negative influence on mental health through sports participation.

### Social climate (organizational and community levels)

#### Social climate at the sports club/location

The broader social climate of the sports setting is created by the people within the setting, such as coaches, managers, parents, and the children and adolescents themselves. When sport is performed in an environment that has the *potential to challenge* children and adolescents, but where they also feel *safe*, it provides them with an ideal place to learn new skills and abilities, while simultaneously succeeding and failing in a *nurturing environment*. This type of environment was reported to enhance mental health in grassroots sports [52, 54, 56, 67, 72]. On the other hand, a social climate that focuses *too much on competition or performance*, whether in grassroots or elite sports, can have the opposite effect by enhancing internal pressure to perform (see also: individual mechanisms) and, consequently, stress and negative emotions among children and adolescents [39, 52, 56, 59, 71].

### Summary of results: a socio-ecological model

**Fig. 2:**
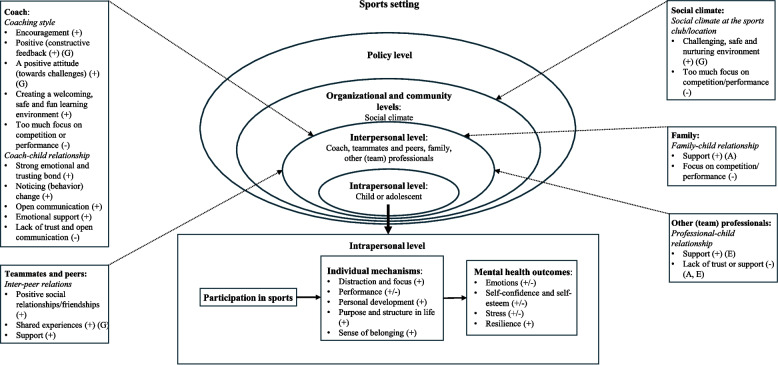
Summary of results in a socio-ecological model*. * In the figure, the findings are organized within a socio-ecological model. Mental health outcomes, along with individual mechanisms, are depicted at the intrapersonal level. The relationship between sports participation and mental health outcomes is influenced by factors operating across multiple levels of the model: the interpersonal, organizational and community, and policy levels. + represents a positive mental health outcome or positive influence on mental health;- represents a negative mental health outcome or negative influence on mental health. When factors apply to a specific subgroup, this is indicated as A (adolescents), G (grassroots sports), or E (elite sports). These nuanced differences are not shown for the individual mechanisms or mental health outcomes

In Fig. 2, the results are summarized in a socio-ecological model [6, 20, 21, 34, 35]. The child or adolescent is placed at the intrapersonal level (first level in the model). It is shown that a young individual who engages in sport can have different mental health outcomes through the individual mechanisms described in this review. However, the relationship between sports participation and mental health outcomes is influenced by interactions with factors at the interpersonal, organizational and community, and policy levels. From the current review, interactions with the interpersonal level (i.e., coaches, teammates/peers, family, other (team) professionals; the second level in the model), and to a lesser extent the organizational and community levels (i.e., the broader social climate; the third level in the model), appeared important. Main themes regarding the most distal level of the social-ecological model (i.e., the policy level; the fourth level in the model) were not identified from the included studies. Policy-level factors might be less visible or salient to children and adolescents and could also exert indirect influences through other levels of the model (e.g., influencing coaching style at the interpersonal level; see Discussion). In line with the socio-ecological model, which reflects the complex, multilevel interactions among intrapersonal, interpersonal, organizational, community, and policy factors, the policy level is nevertheless included. Although caution should be exercised in making broad assumptions regarding relationships, the links in this model between outcomes, individual mechanisms, and factors in the sports setting are based on multiple authors’ interpretations and participant experiences, which strengthens the theoretical groundwork of this model.

## Discussion

This systematic review of 25 qualitative studies showed that sports participation generally had a positive impact on the mental health of children and adolescents, although negative influences were also experienced in certain sports contexts. Individual mechanisms—such as feelings of belonging and internal pressure to perform—played a role in mental health outcomes. Within the broader sports context, interpersonal factors—such as experiences of coach-child and family-child relationships—were key in providing deeper insights into how positive and negative relationships impact the mental health of children and adolescents. The findings will be discussed in more detail below.

### Mental health outcomes (intrapersonal level)

The authors of most of the studies did not provide a clear definition of mental health, and study findings revealed that study participants interpreted and experienced mental health in different ways. This was evident from their description of mental health in relation to sports participation (see also Table 2). Sports participation influenced emotions, self-confidence and self-esteem, levels of stress, and resilience. In this sense, a variety of mental health benefits were experienced. The qualitative findings corroborate quantitative studies [22, 24, 25], by showing that benefits of sports participation in promoting the mental health of children and adolescents were experienced both in individual and team sports (e.g., rugby, skateboarding), grassroots and elite sports, across age groups of children and adolescents, of both genders, and in different countries. However, as noted in previous literature [23, 25], sports participation is not inherently beneficial for mental health. In the current review, negative experiences—such as increased stress and negative emotions—were also reported. These outcomes were largely influenced by interpersonal factors (see also: factors in the sports setting).

### Individual mechanisms (intrapersonal level)

Previous research provides evidence to support that sports participation can positively influence the mental health of children and adolescents through neurobiological, psychosocial, behavioral, and social mechanisms [20, 36, 37, 97]. The findings of the current review align with these insights, particularly regarding the individual mechanisms of ‘personal development’ and ‘sense of belonging’. These can be viewed as examples of psychosocial and social mechanisms, respectively, as sports participation offers the opportunity for mastery and perceived physical competence, and a source of belonging and companionship. Moreover, the current review contributes to a deeper understanding of how sports participation influences the mental health of children and adolescents by identifying additional individual mechanisms that have been less explicitly discussed in earlier sports literature. For instance, sports participation can also serve as a distraction from daily stressors [33, 54, 56, 59, 63, 65–67, 69, 70, 72, 73], offer purpose [52, 54, 55, 66–69, 73] and structure in life [59, 67, 69, 73], and impact mental health through performance-related experiences [33, 39, 52, 53, 55, 57, 59, 60, 62, 63, 65, 67–69, 71, 72]. These findings suggest that interventions should not only focus on stimulating personal development or social connections, but also on strengthening individuals’ sense of coherence as proposed by Antonovsky (1987) [98]. This can be achieved by providing meaning (e.g., defining specific and realistic sports goals), structure and predictability (e.g., offering sports activities multiple times a week to enhance comprehensibility), and manageable challenges for different experience levels (e.g., offering performance opportunities suited to varying skill levels).

Furthermore, it is plausible that participation in sports may influence mental health through a combination of the identified mechanisms. Still, the exact mechanisms through which sports confer mental health outcomes are likely to vary between individuals, sports activities and sports contexts (see also: factors in the sports setting). In this review, only nuanced differences were observed between specific subgroups. For instance, adolescents particularly emphasized the importance of performing well in sports for their mental health, whereas for grassroots participants, certain aspects of personal development, such as increased physical competence and the experience of succeeding, were especially prominent. Moreover, characteristics of sports activities, such as the frequency, intensity, and duration of training, were often not described in the included studies. There is some evidence that children and adolescents who participate more frequently in sports, with a higher intensity or with a longer duration (e.g., started at a younger age or participated for a longer period; see also: interconnections between mental health and sport drop-out) may have better mental health compared to their peers [21–24, 99, 100]. However, studies also show that excessive sports participation—due to the large time commitment and/or possibility of overtraining—may also have detrimental effects on mental health, such as stress and burn-out [23, 25]. In the current review, some children and adolescents mentioned that balancing sports participation with other life demands (e.g., school, friends) was experienced as stressful [33, 59]. Therefore, how characteristics of sports participation—such as frequency, intensity, and duration—influence mental health through these individual mechanisms warrants further exploration.

### Factors in the sports setting

This systematic review provides an in-depth insight into factors in sports settings that influence the relationship between sports participation and mental health in children and adolescents. Previous quantitative studies have provided evidence that participation in team sports may enhance mental health outcomes compared with individual sports activities due to the social nature of the participation [21–23, 25]. Consistent with these findings, the current review identified interpersonal factors as critical in linking sports participation to mental health outcomes. However, it is important to note that many individual sports (e.g., diving, skateboarding) were often practiced in social or group contexts. This suggests that the social context of participation, rather than the type of sport per se, may be key to promoting mental health. This idea is further reinforced by the role of the broader social climate at the organizational and community levels. Yet, apart from the social climate, other organizational, community, and policy-level factors were not commonly perceived as having a direct influence on the relationship between sports participation and mental health. Policy-related factors, in particular, may be less visible or salient to children and adolescents. Instead, they may exert an indirect influence by shaping the social climate, coaching styles, or access to high-quality sports experiences. Many studies lacked detailed descriptions of sports settings, especially regarding community, organizational, and policy-level characteristics. Moreover, some sports activities took place in unorganized settings, where these distal factors may be less visible or less relevant. The recruitment of research participants from multiple sports settings (e.g., different sports clubs) further complicates assessing the influence of the outer levels of the socio-ecological model, and actors at these levels (e.g., sports club managers) were often (with one exception) not included in the research. Notably, three studies included in the review reported that adolescents experienced greater mental health benefits from participating in outdoor sports and nature-based activities, compared to indoor sports. These benefits were linked to increased enjoyment and reduced stress levels [67, 70, 73]. This suggests that distal factors, such as the physical environment at the organizational and community levels, may still play a meaningful role and should not be overlooked. Performing case studies (see, for example, [101]) including actors from multiple levels of a sports setting (e.g., children, adolescents, parents, coaches, and club managers of a specific sports club) could provide valuable insights into how these factors interact and influence mental health.

Overall, most children and adolescents in the reviewed studies participated in sports within social contexts, where interpersonal factors appear to play a more prominent role in supporting mental health than organizational, community, or policy-level factors. Nevertheless, it is important for future research to explore how these dynamics might shift when young people engage in sports independently, without a strong social component. In such cases, distal factors, such as access to natural environments, the availability of safe and well-equipped sports facilities, and funding for quality programming, may become more central to supporting mental health through sports participation.

### Interpersonal level

As previously discussed, interpersonal-level factors play a pivotal role in influencing the mental health of children and adolescents through sports participation. Key influences at this level include coaches, teammates and peers, family members, and other professionals involved with the team.

#### Coach

The current review shows that coaches can play an important role in stimulating the mental health of children and adolescents through sports participation. However, the extent to which coaches perceive themselves as responsible for supporting the mental health of children and adolescents may vary considerably between coaches [57, 61, 64]. Traditionally, the core focus of a sports coach is to develop a young individual’s sports performance [23, 64]. In addition, many coaches may lack the necessary knowledge and training to positively contribute to mental health [57, 61, 64, 102]. Moreover, external pressures, such as performance expectations, winning demands, and administrative responsibilities (e.g., team selection) can lead to coaching behaviors that are damaging to a young individual’s mental health [23, 103]. These issues are highlighted in the current review, particularly in relation to coaching styles that overly emphasize competition or performance, and coach-child relationships characterized by a lack of trust and open communication.

Despite growing recognition of the coach’s potential role in stimulating mental health of children and adolescents [53, 57, 61, 64], existing educational resources for coaches often provide limited practical guidance [104]. In this context, the findings of the current review provide concrete strategies to support coaches in fostering the mental health of children and adolescents. These strategies include adopting a positive coaching style characterized by encouragement and by creating an inclusive, welcoming environment that prioritizes enjoyment and feelings of safety. For children and adolescents participating at the grassroots level, providing positive constructive feedback and maintaining a positive attitude towards challenges are also important. Furthermore, it is crucial that coaches invest time in building strong, trusting relationships with children and adolescents, and encourage open communication. This enables coaches to detect potential changes in mental health and provide appropriate emotional support when needed. These strategies complement and enhance general health-promoting coaching skills (e.g., communication, motivation) and actions specific on the topic of mental health [102, 105]. Furthermore, the strategies complement the expert consensus on coach behaviors that support athlete mental health, particularly those within the domain of primary prevention of mental health issues [104]. To ensure effective implementation, it is essential that such strategies are integrated into coach education and ongoing professional development. Additionally, the development of specific guidelines on this topic would be highly valuable [102]. Such efforts would help embed the promotion of mental health among children and adolescents into everyday coaching practices, making mental health a central component of youth sports participation.

#### Teammates and peers

The current review shows that teammates and peers can positively influence the mental health of children and adolescents through positive relationships and friendships, shared experiences (particularly in grassroots sports), and the provision of social support. The literature provides evidence that connections with other people are sources of a sense of belonging and companionship, which in turn can enhance mental health [37]. In addition, social support, especially perceived emotional support, has been found to directly and positively impact mental health, proving beneficial both during periods of adversity and in more stable times [37]. This was corroborated in the present review, where children and adolescents reported receiving social support in various forms—such as encouragement while learning new sports skills, and emotional support during sports-related failures. Furthermore, people are more likely to feel supported by other group members if they strongly feel connected with the group, and group members are more likely to actually offer another person support if they perceive them as a fellow ingroup member [106]. So, the strong social bonds that children and adolescents develop with teammates and peers through sports participation may be very important to protect and stimulate their mental health. However, the influence of teammates and peers is not always positive. Negative influences such as bullying or hazing—though not reported by participants in the reviewed studies—have been identified in other literature as potential mental health risks [6, 23]. Only two included studies in this review noted that an overemphasis on competition and performance could lead to interpersonal tensions, aggressive behaviors, and heightened negative emotions among team members [67, 71]. Nonetheless, these outcomes did not emerge as dominant themes in the current review. Thus, the potential negative impact of teammates and peer interactions on mental health in sports settings remains an area warranting further investigation (see also: interconnections between mental health and sport drop-out and limitations).

#### Family

The current review highlights that family members, particularly parents, play a significant role in influencing the mental health of children and adolescents through their involvement in sports. This influence can be both positive and negative, depending upon the situation and nature of the family-child relationship. For example, experiencing a loss or underperforming in a game may negatively affect a young individual’s mental health (i.e., via the individual mechanism of unsuccessful performance). However, when parents provide emotional support and encouragement to learn from the experience and improve, they can buffer these negative mental health effects. Interestingly, children generally did not report parental support as a key factor influencing their sports participation or mental health, whereas adolescents frequently emphasized its importance. This difference likely reflects developmental factors: younger children may be less aware of or able to articulate the impact of parental support, focusing primarily on immediate experiences in sport, while adolescents are more capable of recognizing and valuing the emotional and motivational guidance provided by their parents. At the same time, both children and adolescents experienced increased pressure when parents prioritized performance and winning over personal growth, which could exacerbate negative mental health outcomes, for example, if parents expressed disappointment in their child’s performance. The literature supports the dual nature of parental involvement—both supportive and pressuring behaviors have been linked to corresponding positive and negative mental health outcomes [6, 23, 107, 108]. Furthermore, research indicates that the role of parents evolves over time, shifting from primarily facilitating and supporting opportunities for sports participation during childhood to providing more tangible (e.g., financial and time commitments) and emotional support during adolescence, when young individuals often dedicate themselves to one or two sports, engage in higher levels of deliberate practice, and compete in more demanding competitions [103, 107, 108].

Despite their significant influence, parents are often overlooked as key contributors to their children's mental health within sport contexts. In some cases, they are even deliberately excluded from their child's sports experience by coaches and sports organizations [108]. Moreover, there is limited understanding of how parents can proactively foster positive mental health outcomes as part of their child’s sports experiences [108]. Based on the current review, some recommendations can be made to support parents in this role: these include that parents should take time to talk with and actively listen to their children about their sports experiences, and provide understanding and emotional support, particularly as children grow older and the demands and challenges of sport increase. Furthermore, rather than emphasizing competition or performance, parents should support participation, enjoyment, effort, personal improvement, and skill development. Moreover, parents should be made aware of their potential role to positively influence mental health through sports participation, and be offered concrete educational resources and opportunities to engage in this role effectively [108]. These recommendations may apply to other family members as well.

#### Other (team) professionals

Particularly in elite sports settings, additional professionals, such as team managers, sport psychologists, lifestyle coaches, and nutritionists, may be present and can play a role in influencing the mental health of children and adolescents. Children and adolescents reported varying experiences with this formal support in relation to their mental health. In particular, some adolescents have experienced a lack of trust in, and insufficient support from, these professionals. As noted earlier, adolescents often face greater sport demands and challenges than children [103, 107], which may require more individualized and in‑depth support. When professionals are unable to meet these evolving needs, gaps in support become particularly apparent and may, in turn, negatively affect adolescents’ mental health. Furthermore, adolescents are at a developmental stage characterized by greater behavioral autonomy [109], and may be more sensitive to perceived control and judgment from adults than children. This heightened sensitivity may make adolescents particularly affected by the style, approach, or perceived expertise of the professionals supporting them.

In general, professionals’ influence on mental health may depend on their background and expertise. For instance, a sport psychologist may be better positioned to support mental health than a nutritionist. Additionally, the role professionals play can vary depending on their knowledge of mental health and whether they perceive mental health as part of their professional responsibilities. Research among sport psychologists highlights this variability: while some see their role as solely performance-focused, others prioritize mental health within their practice [110]. This dichotomy aligns with the mixed experiences reported by young individuals in this review. Although mental health education for sport psychologists has improved in recent years, training remains insufficient for many to support sports participants' mental health with confidence [110]. This is likely to extend to other professionals working in sports settings. Therefore, a key first step is to enhance education and training—raising awareness among these professionals about the vital role they can play in supporting the mental health of children and adolescents and equipping them to do so appropriately.

### Organizational and community levels

#### Social climate

The broader social climate of the sports setting plays a critical role in shaping the mental health of children and adolescents. This social climate emerges from the dynamic interplay among various actors in the sports setting, including coaches, managers, parents, and the children and adolescents themselves. It is essential that children and adolescents participate in sports in a nurturing environment in which they are challenged, but in which they also feel safe to try new things, to fail and to learn. In this sense, competition can also provide challenging situations through which children and adolescents can develop and, consequently, enhance their mental health. This balance between challenge and support has been particularly emphasized in grassroots sports. However, both in grassroots and elite sports, an overly competitive environment can be detrimental, increasing pressure to perform, and potentially harming children’s and adolescents’ mental health. Therefore, fostering a social climate that is both supportive and appropriately challenging is critical across all levels of sport.

The positive social climate and the interpersonal relationships described earlier between young individuals and their coaches, parents, and peers have many similarities with the principles of Positive Youth Development (PYD) through sport [111]. This alignment is expected, given the interconnection between youth development and mental health, as well as the influence of developmental transitions on the relationship between sports participation and mental health outcomes [6]. Despite these conceptual overlaps, mental health is not typically defined as an explicit outcome within existing PYD-frameworks [111, 112]. Integrating mental health more explicitly into sport-based PYD-models, alongside the factors identified in this review, may enhance the potential of such frameworks to support the holistic development of children and adolescents [112].

### Interconnections between mental health and sport drop-out

While PYD-frameworks highlight the developmental potential of sport, examining the factors that link mental health and sport drop-out offers additional insight into how sports participation can both support or hinder a young individual’s mental health. Many of the same interpersonal factors that promote mental health and growth within PYD also play a crucial role in determining whether young individuals continue participating in sport or decide to leave. Positive and supportive experiences (e.g., friendships, encouragement, social support) can foster both mental health and sustained participation, whereas negative or unsupportive experiences (e.g., lack of support, excessive emphasis on competition or performance) may contribute to poorer mental health and increase the likelihood of discontinuation [113–116].

There is also overlap in individual mechanisms. For example, experiences of personal growth and successful performance can enhance both mental health and continued sports participation, whereas limited personal development or repeated performance failures may have the opposite effect [114–116]. Moreover, research indicates that children and adolescents who drop out of sport tend to experience poorer mental health later in adolescence and adulthood compared with peers who maintain participation [99, 100], and even those who never engaged in youth sports at all [99].

However, most studies, including those reviewed here, primarily focus on children and adolescents who remain active in sport, potentially overlooking the experiences of those who have dropped out. This selection bias may have led to an underrepresentation of young individuals with negative sport experiences or poorer mental health and, consequently, of the factors that hinder mental health through sports participation. Future research should, therefore, aim to better capture the perspectives of former participants and explore how these interconnected interpersonal and intrapersonal factors simultaneously influence both sport engagement and mental health outcomes.

### Children versus adolescents

This systematic review focused on the influence of sports participation on the mental health of children and adolescents. Overall, the findings suggest that individual mechanisms and influencing factors are largely comparable between these age groups, with only some nuanced differences highlighted in the results and discussion sections. However, caution is warranted when interpreting these findings. Children were generally underrepresented in the included studies: only seven studies specifically included children as an interest group, and in just three of these were children active research participants. Moreover, no studies explicitly examined differences between children and adolescents, and when both groups were included, findings were often aggregated into overarching themes. As a result, subtle differences in experiences between children and adolescents may have been overlooked. For example, social support from teammates and peers was reported as important for both groups, but the specific nature and content of this support may change as individuals transition from childhood to adolescence, and across different developmental stages within these age groups. These nuanced shifts were not detectable in the current review.

Furthermore, most studies were cross-sectional in nature or too short in duration to capture possible changes in individual mechanisms or influencing factors over time. As discussed, the role and influence of parents and team professionals may vary across developmental phases, and the same applies to coaches and peers [6, 103, 107, 108]. Therefore, to better capture such differences, longitudinal research is needed that examines the influence of individual mechanisms and broader contextual factors over an extended period. This approach would allow for a more precise understanding of how developmental transitions affect the relationship between sports participation and mental health in children and adolescents.

### Practical implications

Overall, the findings of this systematic review are valuable for sports practitioners, policy makers, and other stakeholders aiming to promote the mental health of children and adolescents through sports participation. The theoretical model presented in Fig. 2 is unique in that it focuses specifically on children and adolescents in sports settings. It integrates mental health outcomes with individual mechanisms and broader contextual factors that influence the relationship between sports participation and mental health. Based on this model, concrete strategies can be developed and implemented—some of which have already been outlined in the discussion. For example, sports clubs and national sports federations can use the proposed model (see Fig. 2) to inform the integration of mental health promotion into coach education. It also offers guidance for recent initiatives, such as the mental health guidelines for community sport, which advocate psychologically safe coaching practices and the well-being of sports participants [27]. For policymakers, the findings underscore the importance of investing in strategies that encourage youth participation in sports and support the creation of mentally healthy sports environments. Crucially, multi-component strategies should be promoted—targeting not only the sports participants, but also coaches, parents, and other team professionals—since most current interventions focus solely on athletes [117]. Further testing and theory building are necessary, particularly concerning the more distal levels of the model (i.e., the organizational, community, and policy levels), and possible changes or shifts in the combination of individual mechanisms and influencing factors across different developmental stages. Also, the links between the model and sport drop-out should be further explored. Moreover, the review also highlights a gap in existing literature: many studies lack a clear theoretical framework and a consistent definition of mental health. Thus, the model and outcomes presented here offer a foundation for future theoretical development and complement the recently proposed systems theory of mental health in recreational sports [28].

### Strengths and limitations

The present review has several strengths. It is the first to systematically synthesize qualitative literature on the topic of sport and mental health in children and adolescents. Importantly, it incorporates the perspectives of multiple actors, including children, adolescents, parents, and coaches, which enhances the credibility and richness of the findings. The quality of studies was overall good, and the review was prospectively registered and conducted in accordance with established reporting guidelines, including PRISMA and ENTREQ [42–44]. Despite these strengths, some limitations should be acknowledged. First, while broad search terms were employed to capture a wide range of relevant studies, some articles may have been missed or excluded, particularly those addressing sports-related experiences that affect mental health without explicitly using the term ‘mental health’. This may have led to an underrepresentation of factors or situations that have a negative influence on the mental health of children and adolescents, such as bullying, hazing, and coaches’ abusive behaviors [6, 23, 103]. Second, the findings are based on studies involving a variety of subgroups, but some groups were overrepresented, such as adolescent males. This imbalance may have influenced the thematic outcomes, and the limited focus on subgroup differences in the original studies further restricts the depth of analysis. This applies not only to children versus adolescents, as noted earlier, but also to boys versus girls. Only a few differences between boys and girls were reported in the included studies, but these did not pertain to the main themes or sub-themes in this review. Girls, for example, experienced more mental health problems in relation to body image and body weight [55, 60], while boys were less open to speak about their mental health problems [38, 60]. Future research should explore these differences in greater detail. Third, most included studies were conducted in high-income countries, particularly Australia (*n* = 12), where mental health promotion through sports participation may receive greater attention [6, 27, 28]. This may limit the generalizability of findings to other sports settings and low- or middle-income countries where this attention is less or lacking. Fourth, most studies did not provide information about how research participants were finally selected and lacked a description of the researcher-research participant relationship. It is possible that individuals with more positive experiences of sports participation and mental health were more likely to participate in the studies, potentially leading to an underreporting of negative experiences. As noted earlier, this selection bias may also mean that those who had already dropped out of sport were underrepresented. These individuals may be more likely to report negative sport experiences or poorer mental health compared to those who remain active. Consequently, current evidence may disproportionately reflect the perspectives of active sports participants rather than those whose disengagement could provide valuable insights into factors that negatively influence mental health, and the links between mental health and sport drop-out. Therefore, including the perspectives of former sports participants in future research is essential. In general, actively engaging vulnerable young individuals in qualitative research presents both methodological and ethical complexities. However, such involvement can provide richer and more authentic insights into their lived experiences, as well as the factors that shape their engagement and mental health within sports settings [118]. Furthermore, the perspectives that researchers bring into a study as insiders and/or outsiders may affect how they analyze, interpret and report the findings [119]. That said, most studies scored highly on the rigorousness of data-analysis and often included a ‘critical friend’ to reflect on the analysis and results. Nonetheless, future studies should provide more detailed descriptions of recruitment strategies and researcher-participant dynamics to better assess their potential influence on results. Finally, some caution is warranted when interpreting relationships between the model’s factors, mechanisms, and outcomes. Multiple influencing factors and mechanisms may interact simultaneously, with variations across individuals, developmental stages, sports activities, and settings. These remain important considerations for future research, including, as noted earlier, further exploration of the linkages between the model and youth sport drop-out.

## Conclusions

This review provides a deeper understanding of how sports participation influences the mental health of children and adolescents, and how broader contextual factors within the sports setting influence this relationship. By synthesizing a previously fragmented body of qualitative literature, the study establishes a more solid knowledge base. The findings are particularly relevant for sports practitioners, policy makers, and other stakeholders interested in enhancing the mental health of children and adolescents through sports participation. While this review reaffirms the potential mental health benefits associated with sports participation, it also highlights that these benefits are highly dependent on interpersonal factors. However, some negative mental health outcomes were also identified and may be underreported. The findings can inform the development of targeted interventions aimed at enhancing the mental health of children and adolescents through sports participation. Future research should focus on testing and refining the theoretical model presented (see Fig. 2), as well as exploring the generalizability of findings across different population groups, developmental stages, sports activities, and sports settings. In addition, the links between the model and sport drop-out should be further explored.

## Supplementary Information

Additional file 1: PRISMA checklist.

Additional file 2: Search strategy per database.

Additional file 3: CASP scoring.

Additional file 4: Results thematic analyses with references.

## Data Availability

Data sharing is not applicable to this article as no datasets were generated or analyzed during the current study.

## Abbreviations

CASP: Critical Appraisal Skills Programme
ENTREQ: Enhancing Transparency in Reporting the synthesis of Qualitative research
PICO: Population, Intervention, Comparator, Outcome
PRISMA: Preferred Reporting Items for Systematic reviews and Meta-Analyses
PYD: Positive Youth Development
WHO: World Health Organization
