# Supplementary material for: How do sports participation and sports settings influence the mental health of children and adolescents? A systematic review of qualitative studies
Source: BMC Public Health. 2025 Dec 18;26:272. doi: 10.1186/s12889-025-25916-x (PMC12822286; doi:10.1186/s12889-025-25916-x)
Supplement: Supplementary file 2 — Additional file 2: Search strategy per database. [file 12889_2025_25916_MOESM2_ESM.pdf]

Search strategy CINAHL (through EBSCOhost)

String 1 Children and adolescents

TI adolescen* OR AB adolescen* OR TI child* OR AB child* OR TI youth* OR AB youth* OR TI teen* OR AB teen* OR TI young OR AB young

String 2 Sport

(MH "Sports+") OR TI sport* OR AB sport* OR (MH "Athletes+") OR TI athlet* OR AB athlet*

String 3 Mental health

(MM "Mental Health") OR TI “mental health” OR AB “mental health” OR TI “mental well*” OR AB “mental well*” OR TI “psychological health” OR AB “psychological health” OR TI “psychological well*” OR AB “psychological well*”

String 4 Qualitative research

TI “action research” OR AB “action research” OR TI “case stud*” OR AB “case stud*” OR TI ethnograph* OR AB ethnograph* OR TI “focus group*” OR AB “focus group*” OR TI “grounded theory” OR AB “grounded theory” OR TI interview* OR AB interview* OR TI mixed-method* OR AB mixed-method* OR TI narrative OR AB narrative OR TI phenomenolog* OR AB phenomenolog* OR TI qualitative OR AB qualitative

Search mode: Proximity
Expanders: None

Search strategy PsycInfo (through Ovid)

String 1 Children and adolescents

(adolescen* OR child* OR youth* OR teen* OR young).ab,ti.

String 2 Sport

exp Sports/ OR "sport*".ab,ti. OR exp Athletes/ OR "athlet*".ab,ti.

String 3 Mental health

exp Mental Health/ OR (mental health OR mental well* OR psychological health OR psychological well*).ab,ti.

String 4 Qualitative research
(action research OR case stud* OR ethnograph* OR focus group* OR grounded theory OR interview* OR mixed-method* OR narrative OR phenomenolog* OR qualitative).ab,ti.

Search strategy PubMed

String 1 Children and adolescents

adolescen*[tiab] OR child*[tiab] OR youth*[tiab] OR teen*[tiab] OR young[tiab]

String 2 Sport

“Sports”[Mesh] OR sport*[tiab] OR “Athletes”[Mesh] OR athlet*[tiab]

String 3 Mental health

“Mental Health”[Mesh] OR “mental health”[tiab] OR “mental well*”[tiab] OR “psychological health”[tiab] OR “psychological well*”[tiab]

String 4 Qualitative research
“action research”[tiab] OR “case stud*”[tiab] OR ethnograph*[tiab] OR “focus group*”[tiab] OR “grounded theory”[tiab] OR interview*[tiab] OR mixed-method*[tiab] OR narrative[tiab] OR phenomenolog*[tiab] OR qualitative[tiab]

Search strategy Scopus

String 1 Children and adolescents

(TITLE-ABS(adolescen*)) OR (TITLE-ABS(child*)) OR (TITLE-ABS(youth*)) OR (TITLE-ABS(teen*)) OR (TITLE-ABS(young))

String 2 Sport

(TITLE-ABS(sport*)) OR (TITLE-ABS(athlet*))

String 3 Mental health

(TITLE-ABS({mental health})) OR (TITLE-ABS("mental well*")) OR (TITLE-ABS({psychological health})) OR (TITLE-ABS("psychological well*"))

String 4 Qualitative research

(TITLE-ABS({action research})) OR (TITLE-ABS("case stud*")) OR (TITLE-ABS(ethnograph*)) OR (TITLE-ABS("focus group*")) OR (TITLE-ABS({grounded theory})) OR (TITLE-ABS(interview*)) OR (TITLE-ABS(mixed-method*)) OR (TITLE-ABS(narrative)) OR (TITLE-ABS(phenomenolog*)) OR (TITLE-ABS(qualitative))

Search strategy SPORTDiscus (through EBSCOhost)

String 1 Children and adolescents

TI adolescen* OR AB adolescen* OR TI child* OR AB child* OR TI youth* OR AB youth* OR TI teen* OR AB teen* OR TI young OR AB young

String 2 Sport

DE "SPORTS" OR TI sport* OR AB sport* OR DE "ATHLETES" OR TI athlet* OR AB athlet*

String 3 Mental health

DE "MENTAL health" OR TI “mental health” OR AB “mental health” OR TI “mental well*” OR AB “mental well*” OR TI “psychological health” OR AB “psychological health” OR TI “psychological well*” OR AB “psychological well*”

String 4 Qualitative research

TI “action research” OR AB “action research” OR TI “case stud*” OR AB “case stud*” OR TI ethnograph* OR AB ethnograph* OR TI “focus group*” OR AB “focus group*” OR TI “grounded theory” OR AB “grounded theory” OR TI interview* OR AB interview* OR TI mixed-method* OR AB mixed-method* OR TI narrative OR AB narrative OR TI phenomenolog* OR AB phenomenolog* OR TI qualitative OR AB qualitative

Search mode: Boolean/Phrase (proximity)
Expanders: None

Search strategy Web of Science (through Clarivate)

String 1 Children and adolescents

TI=(adolescen*) OR AB=(adolescen*) OR TI=(child*) OR AB=(child*) OR TI=(youth*) OR AB=(youth*) OR TI=(teen*) OR AB=(teen*) OR TI=(young) OR AB=(young)

String 2 Sport

TI=(sport*) OR AB=(sport*) OR TI=(athlet*) OR AB=(athlet*)

String 3 Mental health

TI=("mental health") OR AB=("mental health") OR TI=("mental well*") OR AB=("mental well*") OR TI=("psychological health") OR AB=("psychological health") OR TI=("psychological well*") OR AB=("psychological well*")

String 4 Qualitative research

TI=("action research") OR AB=("action research") OR TI=("case stud*") OR AB=("case stud*") OR TI=(ethnograph*) OR AB=(ethnograph*) OR TI=("focus group*") OR AB=("focus group*") OR TI=("grounded theory") OR AB=("grounded theory") OR TI=(interview*) OR AB=(interview*) OR TI=(mixed-method*) OR AB=(mixed-method*) OR TI=(narrative) OR AB=(narrative) OR TI=(phenomenolog*) OR AB=(phenomenolog*) OR TI=(qualitative) OR AB=(qualitative)
