# Supplementary material for: How do sports participation and sports settings influence the mental health of children and adolescents? A systematic review of qualitative studies
Source: BMC Public Health. 2025 Dec 18;26:272. doi: 10.1186/s12889-025-25916-x (PMC12822286; doi:10.1186/s12889-025-25916-x)
Supplement: Supplementary file 3 — Additional file 3: CASP scoring. [file 12889_2025_25916_MOESM3_ESM.docx]

|  | **CASP criteria** | | | | | | | | | |
| --- | --- | --- | --- | --- | --- | --- | --- | --- | --- | --- |
| **Author(s) (year of study) (reference)** | A1. Was there a clear statement of the aims of the research? | A2. Is a qualitative methodology appropriate? | A3. Was the research design appropriate to address the aims of the research? | A4. Was the recruitment strategy appropriate to the aims of the research? | A5. Was the data collected in a way that addressed the research issue? | A6. Has the relationship between researcher and participants been adequately considered? | B7. Have ethical issues been taken into consideration? | B8. Was the data analysis sufficiently rigorous? | B9. Is there a clear statement of findings? | C10. How valuable is the research? |
| Appelqvist-Schmidlechner et al. (2023) (52) | Yes | Yes | Yes | Can't tell | Yes | No | Yes | Yes | Yes | Yes |
| Brown et al. (2017) (53) | Yes | Yes | Can't tell | Can't tell | Yes | No | Can't tell | Yes | Yes | Yes |
| Caperchione et al. (2022) (54) | Yes | Yes | Yes | Can't tell | Yes | No | Yes | Yes | Yes | Yes |
| Coyle et al. (2017) (55) | Yes | Yes | Yes | Can't tell | Yes | Can't tell | Yes | Yes | Yes | Can't tell |
| Drummond et al. (2022) (56) | Yes | Yes | Can't tell | Can't tell | Can't tell | No | Yes | Can't tell | Can't tell | Yes |
| Elliott et al. (2024) (38) | Yes | Yes | Yes | Can't tell | Yes | Can't tell | Yes | Yes | Yes | Yes |
| Ferguson et al. (2019) (57) | Yes | Yes | Yes | Can't tell | Yes | No | Yes | Yes | Yes | Yes |
| Gervis & Dunn (2004) (58) | Yes | Yes | Can't tell | Can't tell | Yes | No | Can't tell | Can't tell | Yes | Can't tell |
| Gotfredsen et al. (2020) (59) | Yes | Yes | Yes | Can't tell | Yes | Can't tell | Yes | Can't tell | Yes | Yes |
| Gulliver et al. (2012) (60) | Yes | Yes | Yes | Can't tell | Yes | No | Yes | Can't tell | Yes | Yes |
| Hurley et al. (2017) (39) | Yes | Yes | Yes | Can't tell | Yes | No | Can't tell | Yes | Yes | Yes |
| Lebrun et al. (2020) (61) | Yes | Yes | Yes | Can't tell | Yes | No | Yes | Yes | Yes | Yes |
| Marsters & Tiatia-Seath (2019) (62) | Yes | Yes | Yes | Can't tell | Yes | Yes | Yes | Yes | Yes | Yes |
| Marsters et al. (2020) (63) | Yes | Yes | Yes | Can't tell | Yes | Yes | Yes | Yes | Yes | Yes |
| Mazzer & Rickwood (2015) (64) | Yes | Yes | Can't tell | Can't tell | Yes | No | Yes | Yes | Yes | Yes |
| Morrongiello et al. (2024) (65) | Yes | Yes | Can't tell | Can't tell | Yes | Yes | Yes | Yes | Yes | Yes |
| Moss et al. (2020) (66) | Yes | Yes | Yes | Yes | Yes | No | Yes | Yes | Yes | Yes |
| Murphy et al. (2022) (67) | Yes | Yes | Can't tell | Can't tell | Yes | No | Yes | Yes | Yes | Can't tell |
| Page & Coetzee (2021) (68) | Yes | Yes | Yes | Can't tell | Yes | No | Yes | Can't tell | Yes | Yes |
| Pittaway & Dantas (2022) (69) | Yes | Yes | Yes | Can't tell | Yes | Yes | Yes | Yes | Yes | Yes |
| Swann et al. (2018) (33) | Yes | Yes | Yes | Can't tell | Yes | No | Yes | Yes | Yes | Yes |
| Toyama et al. (2022) (70) | Yes | Yes | Can't tell | Can't tell | Yes | No | Yes | Yes | Yes | Yes |
| Vaughan et al. (2022) (71) | Yes | Yes | Yes | Can't tell | Yes | Can't tell | Yes | Yes | Yes | Yes |
| White & Bennie (2015) (72) | Yes | Yes | Yes | Can't tell | Yes | Yes | Yes | Yes | Yes | Yes |
|  | **CASP criteria** | | | | | | | | | |
| **Author(s) (year of study) (reference)** | A1. Was there a clear statement of the aims of the research? | A2. Is a qualitative methodology appropriate? | A3. Was the research design appropriate to address the aims of the research? | A4. Was the recruitment strategy appropriate to the aims of the research? | A5. Was the data collected in a way that addressed the research issue? | A6. Has the relationship between researcher and participants been adequately considered? | B7. Have ethical issues been taken into consideration? | B8. Was the data analysis sufficiently rigorous? | B9. Is there a clear statement of findings? | C10. How valuable is the research? |
| Williams et al. (2013) (73) | Yes | Yes | Yes | Can't tell | Yes | Can't tell | Yes | Yes | Yes | Yes |
