# Supplementary material for: How do sports participation and sports settings influence the mental health of children and adolescents? A systematic review of qualitative studies
Source: BMC Public Health. 2025 Dec 18;26:272. doi: 10.1186/s12889-025-25916-x (PMC12822286; doi:10.1186/s12889-025-25916-x)
Supplement: Supplementary file 4 — Additional file 4: Results thematic analyses with references. [file 12889_2025_25916_MOESM4_ESM.docx]

**Table Results of the thematic analyses with references**

| **Main theme*** | **Sub-theme* (specific subgroup)**** | **References** |
| --- | --- | --- |
| **Changes in mental health outcomes** | | |
| ***Intrapersonal level - Child or adolescent*** | | |
| *Emotions* | Positive emotions through sports participation (+) | Appelqvist-Schmidlechner et al., 2023; Caperchione et al., 2022; Coyle et al., 2017; Gotfredsen et al., 2020; Hurley et al., 2017; Marsters & Tiatia-Seath, 2019; Morrongiello et al., 2024; Moss et al., 2020; Murphy et al., 2022; Page & Coetzee, 2021; Pittaway & Dantas, 2022; Swann et al., 2018; White & Bennie, 2015; Williams et al., 2013 |
|  | Negative emotions through sports participation (-) | Coyle et al., 2017; Ferguson et al., 2019; Gervis & Dunn, 2004; Gulliver et al., 2012; Marsters et al., 2020; Murphy et al., 2022; Swann et al., 2018; Vaughan et al., 2022; Williams et al., 2013 |
|  | Learning to regulate negative emotions (+) (G) | Appelqvist-Schmidlechner et al., 2023; Gotfredsen et al., 2020; Morrongiello et al., 2024; Toyama et al., 2022; White & Bennie, 2015 |
| *Self-confidence and self-esteem* | Increased self-confidence or self-esteem (+) | Appelqvist-Schmidlechner et al., 2023; Caperchione et al., 2022; Ferguson et al., 2019; Marsters et al., 2020; Morrongiello et al., 2024; Murphy et al., 2022; Page & Coetzee, 2021; White & Bennie, 2015; Williams et al., 2013 |
|  | Decreased self-confidence or self-esteem (-) | Appelqvist-Schmidlechner et al., 2023; Brown et al., 2017; Drummond et al., 2022; Gervis & Dunn, 2004 |
| *Stress* | Relaxation (+) | Appelqvist-Schmidlechner et al., 2023; Gotfredsen et al., 2020; Moss et al., 2020; Murphy et al. 2022; Pittaway & Dantas, 2022; Swann et al., 2018; Toyama et al., 2022; White & Bennie, 2015; Williams et al., 2013 |
|  | Stress through sports participation (-) | Coyle et al., 2020; Gotfredsen et al., 2020; Marsters et al., 2020; Swann et al., 2018; White & Bennie, 2015 |
| *Resilience* | Learning to handle setbacks (+) (G) | Appelqvist-Schmidlechner et al., 2023; Caperchione et al., 2022; Ferguson et al., 2019; Murphy et al., 2022; Pittaway & Dantas, 2022; White & Bennie, 2015 |
| **Individual mechanisms** | | |
| ***Intrapersonal level - Child or adolescent*** | | |
| *Distraction and focus (+)* |  | Caperchione et al., 2022; Drummond et al., 2022; Gotfredsen et al., 2020; Marsters et al., 2020; Morrongiello et al., 2024; Moss et al., 2020; Murphy et al., 2022; Pittaway & Dantas, 2022; Swann et al., 2018; Toyama et al., 2022; White & Bennie, 2015; Williams et al., 2013 |
| *Performance* | Performing well (+) (A) | Coyle et al., 2017; Marsters & Tiatia-Seath, 2019; Marsters et al., 2020; Murphy et al., 2022; Page & Coetzee, 2021; Pittaway & Dantas, 2022; Swann et al., 2018 |
|  | Feeling pressure to perform (-) | Brown et al., 2017; Coyle et al., 2017; Ferguson et al., 2019; Gotfredsen et al., 2020; Gulliver et al., 2012; Hurley et al., 2017; Marsters & Tiatia-Seath, 2019; Marsters et al., 2020; Murphy et al., 2022; Swann et al., 2018; Vaughan et al., 2022 |
|  | Unsuccessful performance and (fear of) injury (-) | Appelqvist-Schmidlechner et al., 2023; Coyle et al., 2017; Gotfredsen et al., 2020; Gulliver et al., 2012; Hurley et al., 2017; Marsters & Tiatia-Seath, 2019; Marsters et al., 2020; Morrongiello et al., 2024; Murphy et al., 2022; Swann et al., 2018; Vaughan et al., 2022; White & Bennie, 2015 |
| *Personal development* | Improving oneself (+) (A) | Coyle et al., 2017; Ferguson et al., 2019; Marsters & Tiatia-Seath, 2019; Murphy et al., 2022; Swann et al., 2018 |
|  | Physical competence in sports (+) (G) | Appelqvist-Schmidlechner et al., 2023; Ferguson et al., 2019; Morrongiello et al., 2024; Murphy et al., 2022 |
|  | Experiences of succeeding (+) (G) | Appelqvist-Schmidlechner et al., 2023; Caperchione et al., 2022; Morrongiello et al., 2024; Murphy et al., 2022; White & Bennie, 2015 |
|  | Learning life skills through sports (+) | Appelqvist-Schmidlechner et al., 2023; Ferguson et al., 2019; Hurley et al., 2017; Murphy et al., 2022; Swann et al., 2018; White & Bennie, 2015 |
| *Purpose and structure in life* | Purpose or meaning (+) | Appelqvist-Schmidlechner et al., 2023; Caperchione et al., 2022; Coyle et al., 2017; Moss et al., 2020; Murphy et al., 2022; Page & Coetzee, 2021; Pittaway & Dantas, 2022; Williams et al., 2013 |
|  | Providing structure (+) (A, G) | Gotfredsen et al., 2020; Murphy et al., 2022; Pittaway & Dantas, 2022; Williams et al., 2013 |
| *Sense of belonging (+)* |  | Appelqvist-Schmidlechner et al., 2023; Caperchione et al., 2022; Drummond et al., 2022; Ferguson et al., 2019; Gotfredsen et al., 2020; Marsters et al., 2020; Morrongiello et al., 2024; Moss et al., 2020; Murphy et al., 2022; Toyama et al., 2022; White & Bennie, 2015 |
| **Factors in the sports setting** | | |
| ***Interpersonal level - Coach*** | | |
| *Coaching style* | Encouragement (+) | Brown et al., 2017; Ferguson et al., 2019; Mazzer & Rickwood, 2015; White & Bennie, 2015; Williams et al., 2013 |
|  | Positive (constructive) feedback (+) (G) | Appelqvist-Schmidlechner et al., 2023; Drummond et al., 2022; Ferguson et al., 2019; White & Bennie, 2015; Williams et al., 2013 |
|  | A positive attitude (towards challenges) (+) (G) | Brown et al., 2017; Ferguson et al., 2019; White & Bennie, 2015 |
|  | Creating a welcoming, safe and fun learning environment (+) | Appelqvist-Schmidlechner et al., 2023; Ferguson et al., 2019; Mazzer & Rickwood, 2015; Swann et al., 2018; Williams et al., 2013 |
|  | Too much focus on competition or performance (-) | Brown et al., 2017; Gervis & Dunn, 2004; Gulliver et al., 2012; Murphy et al., 2022; Swann et al., 2018; Vaughan et al., 2022 |
| *Coach-child relationship* | Strong emotional and trusting bond (+) | Appelqvist-Schmidlechner et al., 2023; Brown et al., 2017; Ferguson et al., 2019; Mazzer & Rickwood, 2015; Swann et al., 2018; White & Bennie, 2015 |
|  | Noticing (behavior) change (+) | Brown et al., 2017; Ferguson et al., 2019; Gulliver et al., 2012; Lebrun et al., 2020; Mazzer & Rickwood, 2015 |
|  | Open communication (+) | Brown et al., 2017; Coyle et al., 2017; Drummond et al., 2022; Elliot et al., 2024; Ferguson et al., 2019; Gulliver et al., 2012; Lebrun et al., 2020; Mazzer & Rickwood, 2015; Swann et al., 2018; White & Bennie, 2015 |
|  | Emotional support (+) | Appelqvist-Schmidlechner et al., 2023; Brown et al., 2017; Coyle et al., 2017; Hurley et al., 2017; Lebrun et al., 2020; Mazzer & Rickwood, 2015; Swann et al., 2018; White & Bennie, 2015 |
|  | Lack of trust and open communication (-) | Drummond et al., 2022; Elliot et al., 2024; Ferguson et al., 2019; Gulliver et al., 2012; Lebrun et al., 2020; Swann et al., 2018 |
| ***Interpersonal level - Teammates and peers*** | | |
| *Inter-peer relations* | Positive social relationships/friendships (+) | Appelqvist-Schmidlechner et al., 2023; Drummond et al., 2022; Ferguson et al., 2019; Gotfredsen et al., 2020; Marsters et al., 2020; Morrongiello et al., 2024; Moss et al., 2020; Murphy et al., 2022; Page & Coetzee, 2021; Pittaway & Dantas, 2022; Swann et al., 2018; Toyama et al., 2022; White & Bennie, 2015 |
|  | Shared experiences (+) (G) | Gotfredsen et al., 2020; Morrongiello et al., 2024; Murphy et al., 2022; White & Bennie, 2015 |
|  | Support (+) | Appelqvist-Schmidlechner et al., 2023; Brown et al., 2017; Coyle et al., 2017; Drummond et al., 2022; Gotfredsen et al., 2020; Gulliver et al., 2012; Hurley et al., 2017; Marsters et al., 2020; Morrongiello et al., 2024; Murphy et al., 2022; Swann et al., 2018; Toyama et al., 2022; White & Bennie, 2015 |
| ***Interpersonal level - Family*** | | |
| *Family-child relationship* | Support (+) (A) | Brown et al., 2017; Coyle et al., 2017; Hurley et al., 2017; Marsters & Tiatia-Seath, 2019; Marsters et al., 2020; Swann et al., 2018 |
|  | Focus on competition/performance (-) | Ferguson et al., 2019; Gulliver et al., 2012; Marsters et al., 2020; Vaughan et al., 2022 |
| ***Interpersonal level - Other (team) professionals*** | | |
| *Professional-child relationship* | Support (+) (E) | Brown et al., 2017; Coyle et al., 2017; Elliot et al., 2024; Gulliver et al., 2012; Lebrun et al., 2020; Marsters et al., 2020 |
|  | Lack of trust or support (-) (A, E) | Coyle et al., 2017; Elliot et al., 2024; Gulliver et al., 2012 |
| ***Organizational and community levels - Social climate*** | | |
| *Social climate at the sports club/location* | Challenging, safe and nurturing environment (+) (G) | Appelqvist-Schmidlechner et al., 2023; Caperchione et al., 2022; Drummond et al., 2022; Murphy et al., 2022; White & Bennie, 2015 |
|  | Too much focus on competition/performance (-) | Appelqvist-Schmidlechner et al., 2023; Drummond et al., 2022; Gotfredsen et al., 2020; Hurley et al., 2017; Vaughan et al., 2022 |

* + = positive mental health outcome or positive influence on mental health; - = negative mental health outcome or negative influence on mental health

** A = adolescents; G = grassroots sports; E = elite sports
